# *Trichoderma lixii* (IIIM-B4), an endophyte of *Bacopa monnieri* L. producing peptaibols

**DOI:** 10.1186/s12866-019-1477-8

**Published:** 2019-05-16

**Authors:** Meenu Katoch, Deepika Singh, Kamal K. Kapoor, R. A. Vishwakarma

**Affiliations:** 1 Microbial Biotechnology Division, Indian Institute of Integrative Medicine, Canal Road, Jammu, 180001 India; 2 Quality Control and Quality Assurance Division, Indian Institute of Integrative Medicine, Canal Road, Jammu, 180001 India; 3 Department of Chemistry, University of Jammu, Jammu, 180001 India; 4 Medicinal Chemistry Division, Indian Institute of Integrative Medicine, Jammu, India

**Keywords:** Intact cell mass spectrometry, *Bacopa monnieri*, Optimization, Peptaibols, Matrix assisted laser desorption/ionisation-time of flight (MALDI-TOF) mass spectrometer

## Abstract

**Background:**

Exploration of microbes isolated from north western Himalayas for bioactive natural products.

**Results:**

A strain of *Trichoderma lixii* (IIIM-B4) was isolated from *Bacopa monnieri* L. The ITS based rDNA gene sequence of strain IIIM-B4 displayed 99% sequence similarity with different *Trichoderma harzianum* species complex. The highest score was displayed for *Hypocrea lixii* strain FJ462763 followed by *H. nigricans* strain NBRC31285, *Trichoderma lixii* strain CBS 110080, *T. afroharzianum* strain CBS124620 and *Trichoderma guizhouense* BPI:GJS 08135 respectively. Position of *T. lixii* (IIIM-B4) in phylogenetic tree suggested separate identity of the strain. Microbial dynamics of *T. lixii* (IIIM-B4) was investigated for small peptides. Medium to long chain length peptaibols of 11 residue (Group A), 14 residue (Group B) and 17 residue (Group C) were identified using Matrix Assisted Laser Desorption/Ionization-Time of Flight (MALDI-TOF) mass spectrometer. Optimization is undeniably a desideratum for maximized production of desirable metabolites from microbial strain. Here optimization studies were carried out on *T. lixii* (IIIM-B4) using different growth media through Intact Cell Mass Spectrometry (ICMS). A multifold increase was obtained in production of 11 residue peptaibols using rose bengal medium. Out of these, one of them named as Tribacopin AV was isolated and sequenced through mass studied. It was found novel as having unique sequence Ac-Gly-Leu-Leu-Leu-Ala-Leu-Pro-Leu-Aib-Val-Gln-OH. It was found to have antifungal activity against *Candida albicans* (25 μg/mL MIC).

**Conclusion:**

In this study, we isolated a strain of *T. lixii* (IIIM-B4) producing medium and long chain peptaibols. One of them named as Tribacopin AV was found novel as having unique sequence Ac-Gly-Leu-Leu-Leu-Ala-Leu-Pro-Leu-Aib-Val-Gln-OH, which had antifungal properties.

**Electronic supplementary material:**

The online version of this article (10.1186/s12866-019-1477-8) contains supplementary material, which is available to authorized users.

## Background

Endophytic microorganisms have immense potential for producing diverse array of small novel secondary metabolites with key biological activities [1–3]. These metabolites have potential to be used as drugs [4]. The North western Himalayas are bestowed with rich plant and microbial biodiversity. Constant metabolic and ecological interactions happening in the ecosystem implies the biological diversity [5]. This biodiversity can lead to the discovery of new chemical entities. Much of the microbial diversity existing in north western Himalayas is unexplored or underexplored. *B. monnieri* (**L.**) belonging to this region is also unexplored.

*B. monnieri* (**L.**) Pennell (Scrophulariaceae) commonly known as Brahmi, has been used for centuries in Ayurvedic medicine either singly or in combination with other herbs for treating gastrointestinal and neurologic disorders such as Alzheimer’s disease, improving memory, anxiety, attention deficit-hyperactivity disorder (ADHD) etc. [6, 7]. It is also known for curing disorders like anemia, ulcers, leprosy, inflammation, enlarged spleen, ascites and tumors. Prior studies have proved that its medicinal effects have been attributed to saponins (bacosides, bacopasides, bacopasponins) [8]. It also has antidepressant, anti-inflammatory, antioxidant and hepatoprotective properties [9–12]. Because of the medicinal properties of the plant, it was selected for the isolation purpose.

Fungi of the *Trichoderma* genus, notably *Trichoderma harzianum* have been extensively used as biopesticides (against deleterious seed and soil borne pathogens) and biofertilizers [13]. The mechanism of biocontrol includes mycoparasitism, antibiosis, competition and cell wall-lytic enzyme activity, while mechanism of biofertilizer includes enhancement of plant growth, acquisition of soil nutrients and induction of plant defense response respectively [14–18]. During interaction with plant, they produce astonishingly high number and structurally diverse secondary metabolites, which include polyketides, terpenoids, steroids, gliotoxins, gliovirins, peptides, proteins and low molecular weight compounds with antibiotic properties [19–23].

*Trichoderma* is also known for peptaibol production [24, 25]. Varieties of peptaibols have been reported from *Trichoderma* spp. [26–28]. From the pharmacological perspective, peptaibols exhibit variety of bioactivities with claims of antibacterial, antifungal, anticancer, immunosuppressive, antimycoplasmic, antitrypanosomal and wound healing properties, while from agricultural perspective, their role in biocontrol and induced disease resistance has also been reported [27–34]. Thus, they have potential to be used in medicine, agriculture and industry [35, 36].

Various peptaibols have been identified using Intact Cell Mass Spectroscopy (ICMS), a prominent fast analytical technique in biological sciences because sample requirement is minimum, sample preparation is simple (dried solid samples are workable) and automated mass analysis is possible within a short span of time. This technique is already in use for identification of different microbial species/strains, peptaibols from fungi and recently used for monitoring the fermentation process of Penicillin V and for optimizing the peptaibol production [37–41].

The current study reports a new strain of *T. lixii* isolated from *B. monnieri* producing medium and long chain peptaibols identified through ICMS by MALDI TOF mass spectrophotometer and MS/MS studies. It also includes optimization of best possible growth conditions for further scale up and isolation of novel secondary metabolites.

## Methods

### Reagents

*α*-cyano-4-hydroxycinnamic acid (CHCA), 2,5-dihydroxybenzoic acid (DHB), Trifluroacetic acid (THF) were acquired from Sigma Aldrich, India. Solvents such as acetonitrile, ethyl acetate, dichloromethane and methanol were obtained from Thermo Fisher, India. Media components were purchased from Himedia, India.

### Isolation of a strain of IIIM-B4

Fully matured wild *B. monnieri* plants were collected randomly between March – April, 2013 from Jammu and Kashmir (altitude of about 32.73°N 74.87°E), India as per the Institute’s guidelines and were authenticated by the taxonomist of the Institute based on its characteristic features. The fungal endophytes were isolated from *B. monnieri* using the method described by Strobel and Daisy [42] and Katoch et al. [43, 44]. IIIM-B4, an endophyte isolated from this plant was chosen for this study.

### Morphological, Microscopical and molecular identification of strain IIIM-B4

Strain IIIM-B4 was characterized on the basis of colony and microscopic features such as the structure of conidiophore, conidia and chlamydospore.

It was identified through ITS based DNA sequencing using ITS1 and ITS4 primers [36–39]. The culture has been submitted to Sir R.N. Chopra, Microbial Resource Centre, Jammu, India with accession no MRCJ-512. Fungal mycelia grown in potato dextrose broth for 5 days were filtered through filter paper (Whatman no-3). The mycelium (500 mg) was then crushed in liquid nitrogen to make fine powder and genomic DNA was extracted [45–48]. Briefly, powdered mycelium was transferred to 5 mL of extraction buffer and vortexed thoroughly. The samples were incubated in water bath at 65 °C for 30 min with intermittent mixing. The tubes were centrifuged at 10,000 *g* for 5–10 min followed by extraction of aqueous layer with chloroform:isoamyl alcohol (24,1). Aqueous layer was collected and DNA was precipitated with 2.5–3 V of absolute ethanol in presence of 1/10th volume of sodium acetate. Tubes were inverted slowly to mix the contents and centrifuged at 8000 *g* for 20 min at 4 °C. White/transparent pellets thus obtained were washed with ice cold 70% ethanol followed by air drying. Dried pellets were dissolved in 20 μL of water (molecular biology grade).

The ITS regions of fungi were amplified with universal ITS1 and ITS4 primers using polymerase chain reaction (PCR) [49]. The PCR reaction mix (20 μL) contained 1× PCR buffer containing 15 mM MgCl_2_, 200 mM of each dNTP, forward and reverse primer 10 pmol (Sigma, USA), 10 ng of DNA and 0.1 U Taq DNA polymerase (Promega, US). The PCR conditions consisted of initial denaturation at 95 °C for 5 min followed by 30 cycles of 94 °C for 30 s (denaturation), 55 °C for 1 min (annealing), 72 °C for 1 min (extension) and 72 °C for 10 min (final extension) [46–48]. The PCR products were resolved on 2% (w/v) agarose gel at 80 V followed by purification using gel extraction kit (Qiagen, USA). The purified product was sequenced using Big Dye Terminator sequencing kit (v. 3.1, Applied Biosystems) using an automatic DNA Sequencer (310 Genetic Analyser, Applied Biosystems, Foster city, CA). The obtained sequence was analyzed by BLASTn tool of NCBI [www.ncbi.nlm.nih.gov/Blast.cgi] to identify the strain [50].

Besides, the ITS sequences were compared to a specific database for *Trichoderma* using *Trich* OKEY2 program, which available online from the International Subcommission on *Trichoderma* and *Hypocrea* Taxonomy (ISTH, www.isth.info) [51].

### Phylogenetic evaluation of strain IIIM-B4

For phylogenetic characterization, *T. lixii* (IIIM-B4) sequence (Gene Bank accession number KF6839108) and relevant downloaded sequences (FJ462763, JN943368, NR_131264, NR137304, KP115286, AF057584, KU729029, JQ745258, KC171340, NR130668, AY380909) were aligned using Clustal W-pairwise sequence alignment of the EMBL nucleotide Sequence Database [46–48]. The sequence alignments were trimmed and verified by the MUSCLE (UPGMA) algorithm [52] using MEGA4 software [53]. The phylogenetic tree was reconstructed and the evolutionary history inferred using the Neighbor-Joinng method [54]. The robustness of the internal branches was also assessed with 1000 bootstrap replication [55]. The evolutionary distances were computed using Maximum Composite Likelihood method [56] and were calculated in the units of the number of base substitutions per site.

### Identification of peptaibols production from *T. lixii* (IIIM-B4) through intact cell mass spectrometry by MALDI TOF mass spectrophotometer

The endophytic fungus was grown on potato dextrose for a period of 15 days at 25 ± 2 °C in an incubator (New Brunswicks, USA). One disc (5 mm) from 15-day old fungal culture plate was used as inoculum. Post fifteen days, fungal growth of all five petri-plates was used for the extraction of secondary metabolites separately.

A few milligrams of fungal mycelia (1–5 mg) was scraped from agar petri plates and suspended in acetonitrile/methanol/water (1,1,1). The suspension was homogenized thoroughly followed by centrifugation at 5000 g for 15 min. Ten μL of this solution was mixed well with 10 μL of matrix solution (α-cyano-4-hydroxy cinnamic acid 5 mg/mL in acetonitrile:water 70:30). It was homogenized well and mixed properly by vortexing for 5 min in an eppendorf tube and then centrifuged at 5000 rpm for 5 min. 1 μL of this mixture was then directly spotted onto target wells of 96 or 364 well plate using premixed two layer volume technique and allowed to air dry prior to analysis. For metabolic profiling, Matrix Assisted Laser Desorption/Ionisation-Time of Flight (MALDI-TOF) mass spectrometer was employed. MALDI-TOF mass spectra was obtained on Applied Biosystems 4800 MALDI TOF/TOF analyzer (AB Sciex, Foster city, USA), equipped with Nd:YAG 200 Hz laser. The extraction voltage was set at 20 kV. To avoid the saturation of detector by matrix ions, gated matrix suppression was applied. The instrument was operated in positive ion reflectron mode. Ions were extracted from 10 different regions of same sample spot. Ion extraction from each region was the result of the accumulation of at least 1000 laser shots having 10 subspectra or 100 shots per subspecta. Mass range was from *m/z* 500 to 2500. Bin size was 0.5 ns and band width was set at 500 MHz. The S/N ratio was kept at 100 to reduce ions arising from matrix and their clusters, small peptides and other unknown contaminants. Mass accuracy was set at ±0.05 Da [57, 58]. Further, MS/MS studies using collision ion dissociation through MALDI-TOF/TOF mass spectrometer were carried out to confirm these peptides as peptaibols.

### Evaluation of different growth medium for optimal production of peptaibols

Different growth media were used for optimal production of peptaibols using ICMS technique. The media were i) Potato Dextrose Agar (PDA) ii) malt extract agar iii) yeast extract malt extract agar iv) Sabouraud dextrose v) Oat meal agar, vi) Rose Bengal agar vii) Potato carrot agar viii) Corn meal agar ix) Synthetic medium [Glucose 5 g; Potassium dihydrogen phosphate 0.8 g; Potassium nitrate 0.72 g; Calcium dihydrogen phosphate 0.2 g; Magnesium sulphate 0.5 g; Manganese sulphate 0.01 g; Zinc sulphate 0.01 g; Copper sulphate 0.005 g; Ferrous sulphate 0.001 Agar 15 g; Distilled H_2_O 1 L] x) synthetic medium [Yeast extract 5 g; Sucrose 30 g; Di-potassium ortho-phosphate 2 g; Magnesium sulphate 0.5 g; Potassium chloride 0.5 g; Zinc sulphate 0.01 g; Copper sulphate 0.7 g; Ferrous sulphate 0.01 Agar 15 g; Distilled H_2_O 1 L]. On these media, Growth characteristics vis a vis peptaibols production were evaluated. Extraction of fungal samples was carried out as described before.

### Isolation and characterization of a peptaibol

For isolation of peptaibols, *T. lixii* (IIIM-B4) was grown on rose bengal agar (500 plates) for a period of 15 days at 25 ± 2 °C in an incubator (New Brunswick, USA). One block (0.5 cm) from 10-day old fungal culture was used as inoculum. After fifteen days, fungal growth of all petri-plates was used for the extraction of peptaibols as mentioned before.

Isolation of the peptaibol from the extract was achieved by HPLC (Agilent) using a Waters Spherisorb DDS2 column, 5 μm, 250 × 10 mm. The mobile phases were water acetonitrile with 0.1% formic acid. Flow rate was adjusted to 2 mL/min. Wavelengths used for detection were 214, 220 and 254 nm. Method was %B (acetonitrile with 0.1% formic acid) 60 to 95% in 5 min, then in next 3 min to 80% and keeping it for another 5 min and then till 25 min dropping back to 60% which continued for another 3 min. Further, it was sequenced through mass studied.

### Antimicrobial activity of isolated peptaibol

Pathogens viz. *Bacillus subtilis* (MTCC No. 121), *Staphylococcus aureus* (MTCC No. 737), *Salmonella typhimurium* (MTCC No. 98), *Pseudomonas aeruginosa* (MTCC No. 424), *Escherichia coli* (MTCC No. 118), *Klebsiella pneumoniae* (MTCC No. 109), *Candida albicans* (MTCC No. 183) were procured from Microbial Type Culture Collection (MTCC), Chandigarh (India) and were grown on Muller Hinton agar while *C. albicans* was grown on Yeast extract peptone dextrose agar (YEPD). The antibacterial activity following the CLSI protocol was studied against the test organisms by microdilution assay [43, 47, 48]. Streptomycin and Amphotericin B were used as positive controls for bacterial pathogens (both Gram-positive and Gram-negative) and for *C. albicans* respectively. Bacterial strains were inoculated into Muller Hinton broth (Hi Media Biosciences) and incubated at 37 °C while *C. albicans* was grown into YEPD medium (Hi Media, Biosciences) at 30 °C with shaking (200 rpm) for 16 h. The cells were quantified according to Mc Farland standard turbidity (0.5 equivalent to 1.5 × 10^8^ colony forming units (CFU/mL) and finally diluted to 1.5 × 10^5^ CFU/mL for all pathogens. Different dilutions (1–100 μg/mL, 0.078–10 μg/mL) were prepared from the stock solutions of fungal compound (10 mg/mL in DMSO) and antibiotic (1 mg/mL in water). 150 μL of each concentration of fungal compound and antibiotics were mixed with 50 μL of media containing 4 × 10^4^ bacterial cells (diluted from stock of 1.5 × 10^8^ CFU/mL). Appropriate negative (DMSO-0.5%) and blank controls (virgin media) were used. 96-well plate was incubated overnight at 37 °C in an incubator shaker with 150 rpm.

Activities were expressed in terms of minimum inhibitory concentration (MIC)/ minimum bactericidal concentration (MBC). The lowest concentration with no visible growth after 18–24 h was considered as minimum inhibitory concentration (MIC) [59]. 50 μL mix of the well showing no growth and two preceding wells were plated on MHA and incubated overnight. to find out their static/cidal effect. The lowest concentration of extract which didn’t contain any bacterial growth upon spotting 50 μl of mix on MHA plates after 24 h incubation at 37 °C was referred as the minimum bactericidal concentration (MBC). The assay was replicated thrice.

## Results

### Morphological and molecular characterization of *Trichoderma lixii* (IIIM-B4)

**Fig. 1:**
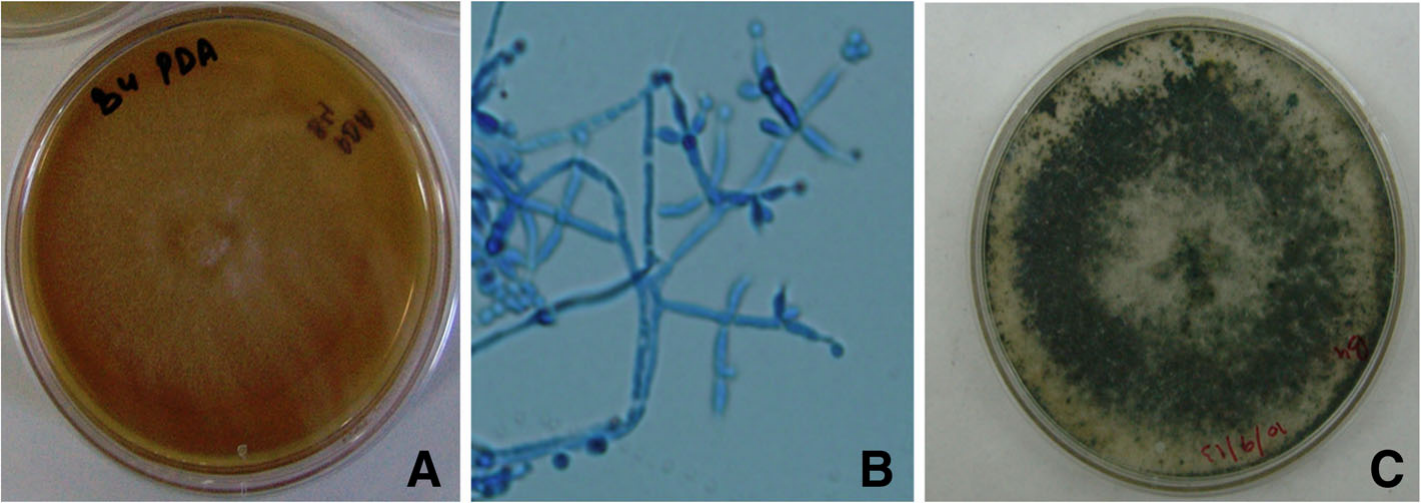
Morphological and microscopical view of *Trichoderma lixii* (IIIM-B4) endophytic fungi associated with *B. monnieri* at 400X magnification **a**) Growth of IIIM-B4 on PDA for 15 days **b**) microscopically view of IIIM-B4 stained with lacto phenol cotton blue **c**) Growth of IIIM-B4 on Rose Bengal medium for 15 days

Endophytic fungi IIIM-B4 was isolated from healthy and symptomless leaves of *B. monnieri* to isolate the bioactive molecule. Endophyte was identified by its characteristic colony morphology and microscopic features (Fig. 1). *T. lixii* (IIIM-B4) grew slowly on PDA medium with white cottony hyphae. Mycelium appeared first smooth, watery white in color, sparse, until floccose aerial mycelium produced. Fifteen-twenty days post incubation; greenish conidia were appeared on the culture plate. In microscopic view, pyramidal conidiophores and effuse conidiation were observed. Ampulliform to flask-shaped phialides were found to have globose or subglobose conidia. In old cultures, terminal or intercalary chlamydospore were also visualized. Growth characteristics of *Trichoderma lixii* B4 on different medium were mentioned in Additional file 1: Table S1 and shown in Additional file 1: Figure S1.

**Fig. 2:**
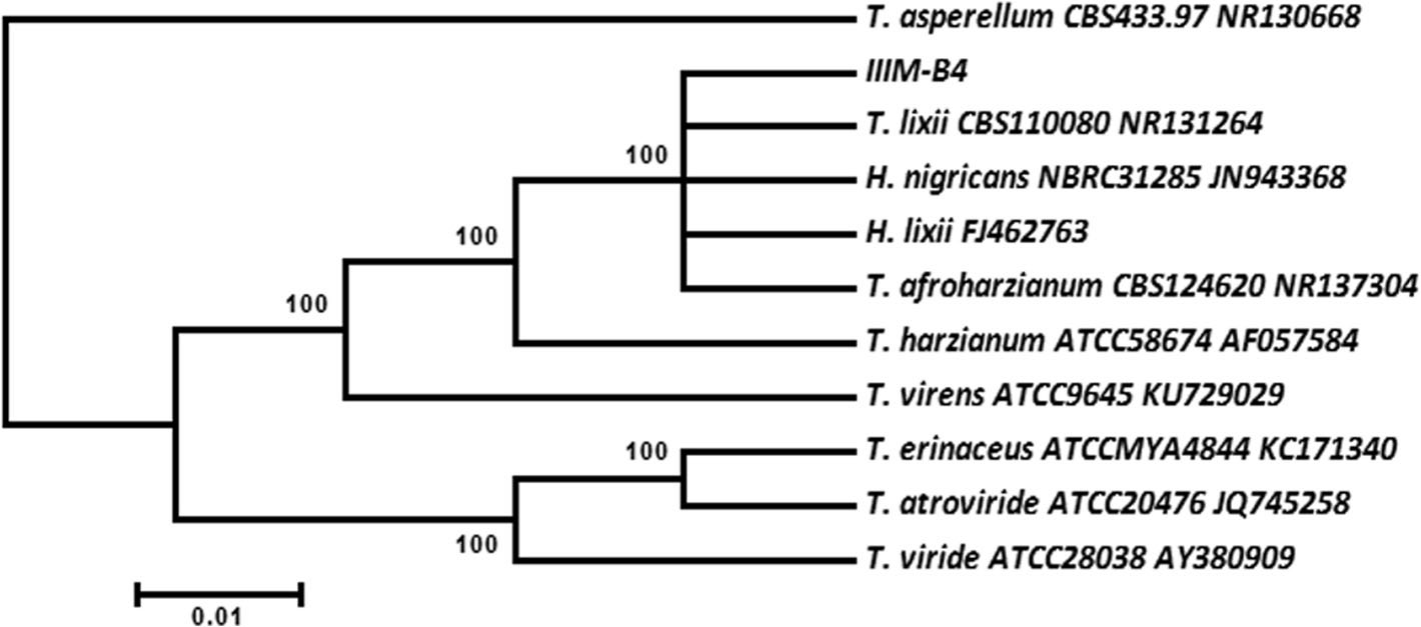
Phylogenetic Tree based on ITS-5.8S rDNA sequence of *Trichoderma lixii* (IIIM-B4) endophytic fungi associated with *B. monnieri* showing the relative position of its close relatives

Further their molecular identification was carried out by ITS based rDNA sequence analysis. Details of the closest sequence homologs of fungal endophyte, their isolation source and their GenBank accession numbers are given in Table 1. Furthermore, ITS sequence data showed that the endophyte is a strain of the *Trichoderma lixii* (Fig. 2). The ITS1 5.8S ITS2 region of ribosomal gene of IIIM-B4 showed a maximum homology of 99% with different *Trichoderma harzianum* species complex. The highest score was displayed for *Hypocrea lixii* strain FJ462763 followed by *H. nigricans* strain NBRC31285, *T. lixii* strain CBS 110080, *T. afroharzianum* strain CBS124620 and *T. guizhouense* BPI:GJS 08135 respectively. To characterize *T. lixii* (IIIM-B4), a phylogenetic tree was constructed which contained two clusters. *T. lixii* (IIIM-B4) was laid down in Cluster I with *Hypocrea lixii, H. nigricans, T. lixii*, *T. afroharzianum*, *T. guizhouense, T. harzianum*, while cluster II contained *T. virens, T. atroviride, T. erinaceus, T. asperellum* and *T. viride.*

**Table 1:** Comparison of the 16 s rRNA gene sequence of IIIM-B4 among isolates of *Streptomyces*

Species	Strain	Similarity (%)	Total score	GenBank accession number
*Hypocrea lixii*	FJ462763	99	1109	FJ462763
*Hypocrea nigricans*	NBRC31285	99	1096	JN943368
*Trichoderma lixii*	CBS 110080	99	1090	NR_131264
*T. afroharzianum*	CBS124620	99	1066	NR137304
*T. guizhouense*	BPI:GJS 08135	99	1040	KP115286
*T. harzianum*	ATCC58674	99	959	AF057584
*T. virens*	ATCC9645	97	998	KU729029
*T. atroviride*	ATCC20476	90	739	JQ745258
*T. erinaceus*	ATCCMYA4844	90	728	KC171340
*T. asperellum*	CBS433.97	89	645	NR130668
*T. viride*	ATCC28038	88	625	AY380909

ITS Sequence of IIIM-B4 was also analyzed through TrichOKEY tool. Reliability of BLAST results was found high. Analysis revealed that it belonged to Genus *Trichoderma,* Section I Lixii - Catoptron Clade, Species *Hypocrea lixii/T. harzianum.* Results were in agreement with the results obtained from GenBank database.

### Identification of peptaibols production from *T. lixii* (IIIM-B4)

**Fig. 3:**
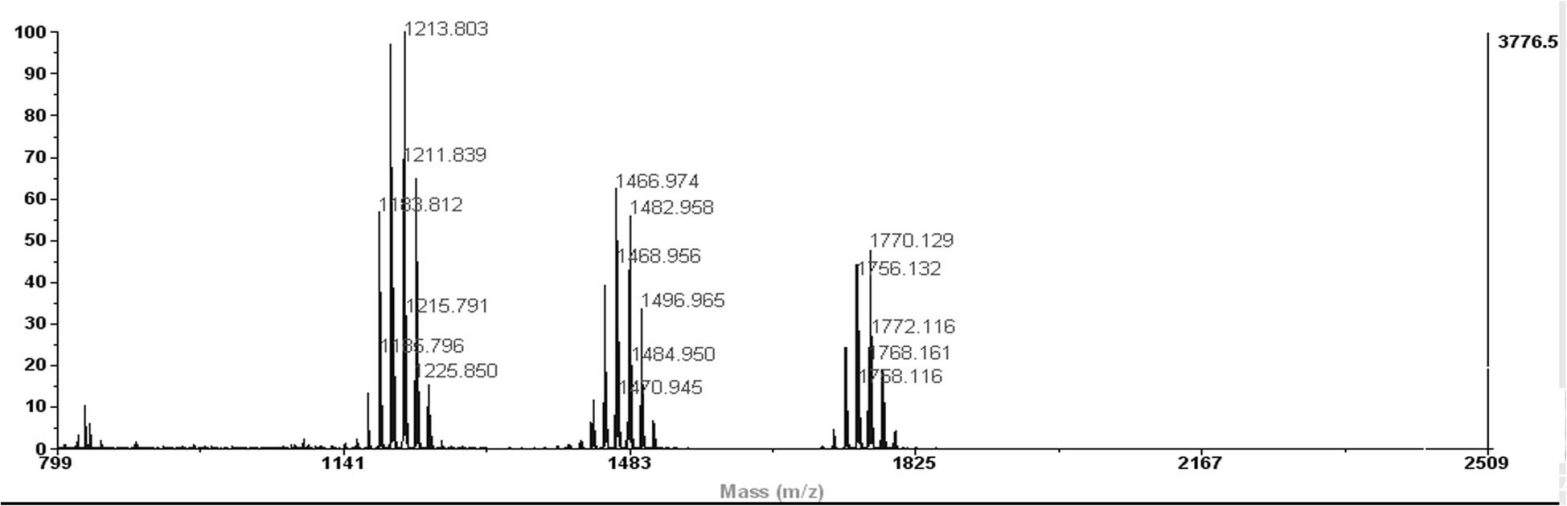
Mass spectral profile of *Trichoderma lixii* (IIIM-B4) obtained through Intact Cell Mass Spectrometry by MALDI TOF mass analyzer indicating three groups of peptaibiotics medium and long chain length

**Fig. 4:**
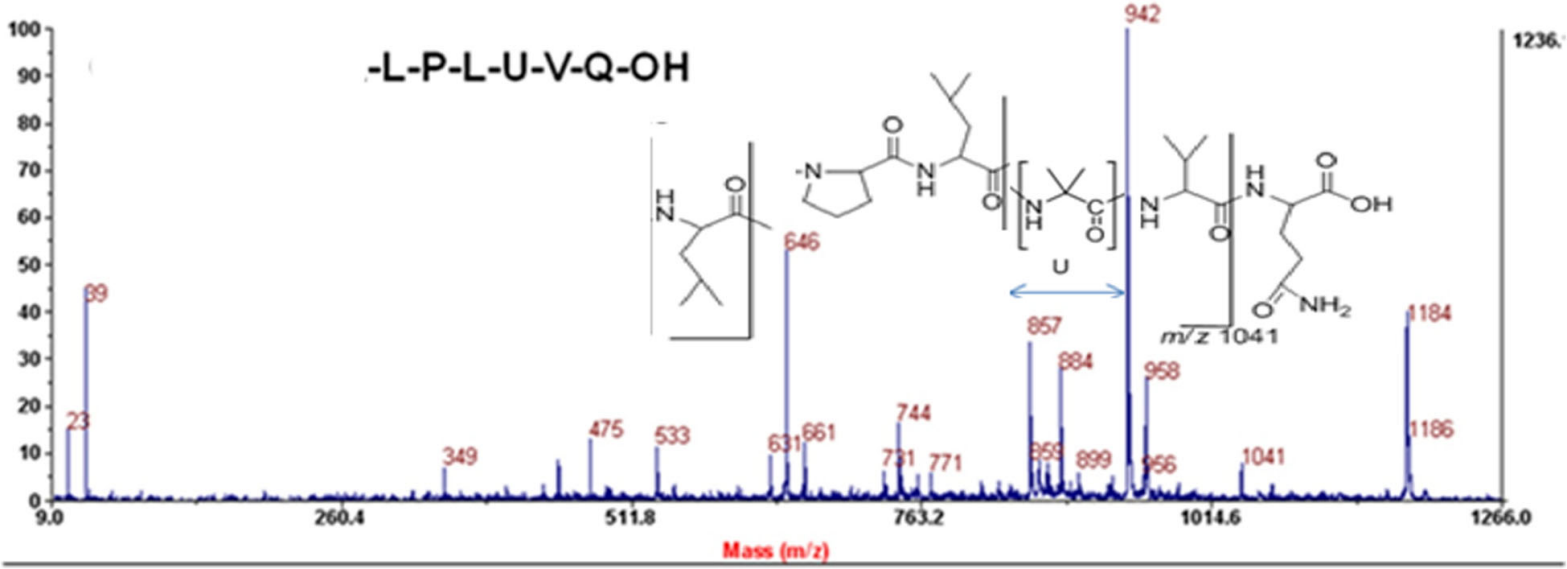
MS/MS spectra showing fragmentation of *m/z* 1185 production by *Trichoderma lixii* (IIIM-B4) for confirming peptaibols

*T. lixii* (IIIM-B4) extract was analyzed through ICMS and was found to be the producer of peptaibols. Illustrious peptaibols spectra of B4 showed characteristic metabolite fingerprints of 11 residue peptaibols (Group A) having mass of *m/z* 1169, 1185, 1198, 1199, 1213, 1215, 1357, 14 residue peptaibols (Group B) having masses as 1466, 1467, 1482, 1484, 1496 and 17 residue peptaibols (Group C) showing masses as 1699, 1756, 1768, 1770 indicating three groups of peptaibols falling in respective medium, medium and long chain length of peptaibols (Fig. 3). MS/MS studies using collision ion dissociation through MALDI-TOF/TOF mass spectrometer were used to characterize these peptides. According to MS/MS spectra, presence of non standard amino acids like *α*-aminoisobutyrate, Aib (U), clearly confirms the identity of peptaibols (Fig. 4). Medium chain peptides having masses *m/z* 1169, 1185, 1198, 1199, 1213, 1215, 1357 and 1466, 1467, 1482, 1484, 1496 belonged to subfamily SF4, where as long chain peptides having mass *m/z* 1699, 1756, 1768, 1770 belonged to subfamily SF1, under physiological pH 7.0.

### Effect of different solid medium on peptaibols production

**Fig. 5:**
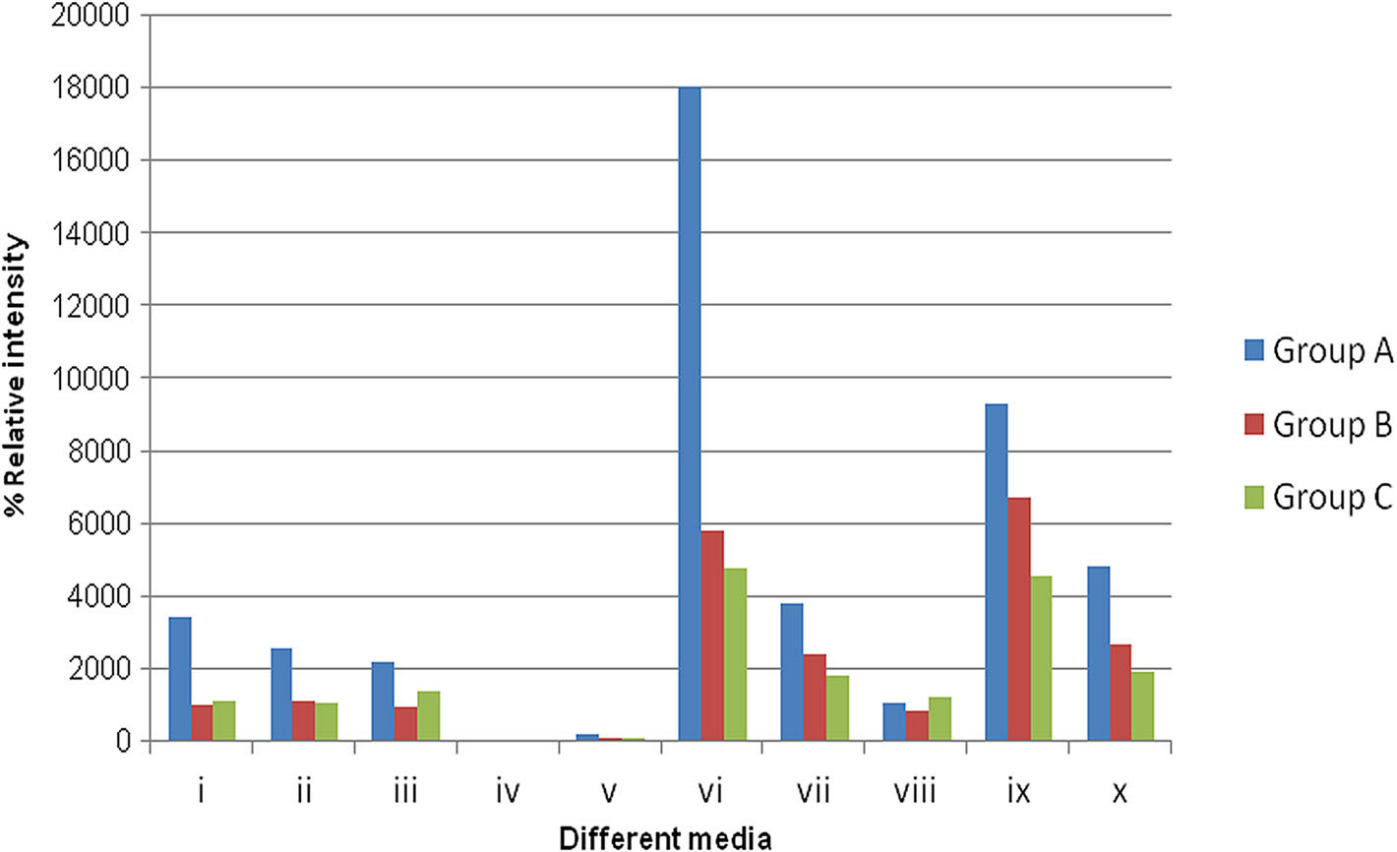
Effect of different media [(i) Potato Dextrose Agar (PDA) (ii) malt extract agar (iii) yeast extract malt extract agar (iv) Sabrauds dextrose agar (v) Oat meat agar (vi) Rose Bengal agar (vii) Potato carrot agar (viii) Corn meal agar (ix) Synthetic medium 1(x) synthetic medium 2] on different group of peptaibiotics production through ICMS (Group A: 11 residue medium chain peptaibiotics in blue; Group B: 14 residue medium chain peptaibiotics in red; Group C: 18 residue long chain peptaibiotics in green)

Peptaibols production was studied through media optimization using ICMS technique (Additional file 1: Figure S2 a’-j). Summation of media optimization studies was shown in Fig. 5, which indicated best quantitative formation of Group A short chain peptaibiotics, named as Tribacopins I-VII in solid media particularly in rose bengal medium upto five fold.

### Isolation and characterization of a peptaibol

**Fig. 6:**
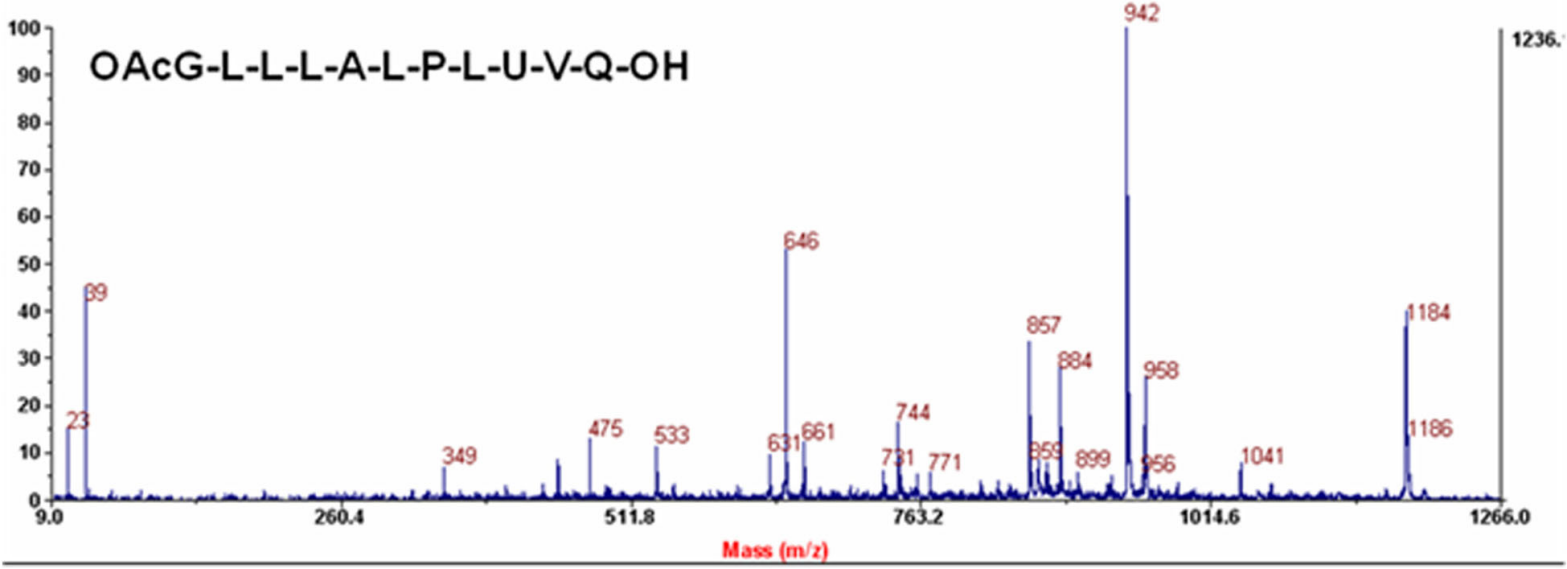
MS/MS spectra showing Sequence of Tribacopin AV

Using the mentioned HPLC program, separation of 11 residue medium chain peptaibols (Fraction A named as Tribacopin AI - AVII) having mass as 1169, 1183, 1184, 1185, 1197, 1198, 1199, 1213 was achieved from long chain peptaibols (Fraction C named as Tribacopin CI-CIII) having mass as 1727, 1729 and 1742 as shown in Additional file 1: Figure S3. Fraction A was further purified and Tribacopin AV having mass as *m/z* 1185 was isolated as shown in Additional file 1: Figure S4. Further, it was sequenced through mass studied. It was found to have a novel sequence (Ac-Gly-Leu-Leu-Leu-Ala-Leu-Pro-Leu-Aib-Val-Gln-OH) as shown in Fig. 6 and Additional file 1: Figure S5.

### Antimicrobial activity of Tribacopin AV

Tribacopin AV was tested for antimicrobial activity and was found to have antifungal activity against *C. albicans* (25 μg/mL MIC) but not active against bacterial pathogens (Additional file 1: Table S2).

## Discussion

Endophytes are ubiquitous in nature. Diverse endophytes have been reported. Endophytes from unique sources are thought to be the producer of unique metabolites. Recent studies have reported isolation of *T. lixii* (IIIM-B4) as endophytes from *B. monnieri* (L.), an ethano-medicinal plant [43, 44]. Phylogenetic characterization based on ITS rDNA sequence suggested that *T. lixii* (IIIM-B4) is a unique strain and showed similarity with *Hypocrea lixii, H. nigricans, T. lixii*, *T. afroharzianum*, *T. guizhouense, T. harzianum* but placed separately in phylogenetic tree. *Hypocrea lixii/nigricans* is a teleomorphic stage of *T. harzianum, a* multispecies complex containing more than thirteen species. Many species including *T. lixii*, *T. afroharzianum*, *T. guizhouense, T. harzianum* belong to *T. harzianum* species complex [60]. Most of the *Trichoderma* spp. including the endophytic ones are peptaibol producers [27]. These peptaibols exhibit a variety of indispensable bioactivities [61]. Metabolite profiling of *T. lixii* (IIIM-B4) using ICMS approach showed intense signals of 11, 14 and 18 residue peptaibols belonging to subfamily 1 and 4. Characterization was done by MS/MS studies using collision ion dissociation through MALDI-TOF/TOF mass spectrometer. According to MS/MS spectra, characteristic feature of presence of non standard amino acids like *α*-aminoisobutyrate, Aib (U), clearly indicated the presence of peptaibols. Similar to present study, Mukherjee et al. [62] also reported 11, 14 and 18 residue peptaibols from *T. virens*, a rhizospheric strain. In contrast to present study, Maddau et al. [27] reported 20 residue peptaibols from an endophyte *T. citrinoviridae* isolated from Cork Oak. Szekeres et al. [31] reported that *Trichoderma/Hypocrea* genus produces the peptaibols of subfamilies 1, 4, 5 and 9.

Optimization is an important aspect for production of desirable metabolite from a microbial strain. Less production of desired metabolites has always been a bottleneck in carrying out purification steps for further studies. While investigating the effect of solid medium on production of peptaibols from *T. lixii* (IIIM-B4), it was astonishing to observe that production of all the peptaibols were not same on different medium. It was found that one or the other desired group of peptaibols was getting adversely affected. Thus, one culture condition does not seem to be advantageous for maximum production of all peptaibols; moreover, it hinders identification of metabolites and their efficient purification. Thus, optimization experiments are important for increasing the yield of desirable secondary metabolites by reducing complexities of natural mixtures to a great extent by favoring the production of one group of metabolite in its respective optimized conditions and giving an edge in process of purification [63]. Bode et al. [64] also reported that small changes in culture conditions affects pronouncedly the production of different metabolites. It has been observed that subsequent storage and sub-culturing under the optimized conditions, further enhances their production.

### Tribacopin AV characterization and its bioactivity

Using the HPLC, Tribacopin AV was isolated having mass *m/z* as 1185. Its sequence was found novel. The new sequence showed connexion with very unique positioning of 2-aminoisobutyric acid (Aib), a hallmark of fungal peptaibols at 9th position, observed across all Trilongins AO, AI, AII a-e, AIII a–d, AIV a-c, Trichorovins I, IV, XIII, XIV, Hypomurocin A-I, Trichobrachins III, VTII, IXa and Trichozin IV. Stimulatingly it was also pragmatic that position of leucine at 3rd and 4th is also concurrent in new sequence of Tribacopin V which is seen in Trilongins AO, AI and in AII a-c Trichobrachins III, Trichorovins VTII, IXa and Hypomurocin A-3. Leu at position 3rd along with Aib at position 9th is seen in Trilongins AII d and in Trilongins A III a-b, Trichobrachins III, Trichorovins XIII, XIV, Hypomurocins A-5, A-5a and Trichorozin IV. Leucine at position 4th along with Aib at position 9th is seen in Trilongins A II e, Trilongins A III c-d, Trichobrachins III, Trichorovins XIII, XIV, Hypomurocins A-5, A-5a and Trichorozin IV [25, 62, 65]. The sequence isolated from *Trichoderma lixii* (IIIM-B4) showed remarkably the unique difference from across all Trilongins, Trichorovins, Trichobrachins, Trichorozins and Hypomurocins that position 5th and 6th is replaced by Alanine-Leucine instead of α, α-di alkyl amino isobutyric acid-Proline [66]. The unique 10th position of Proline observed across all Trilongins, Trichorovins, Trichobrachins, Trichorozins and Hypomurocins is replaced by Valine in Tribacopin AV (Additional file 1: Table S3). This is the first report of Tribacopin AV with novel sequence produced from *T. lixii* (IIIM-B4) isolated from medicinal plant *B. monnieri* L.

Tribacopin AV was analyzed against human bacterial and fungal pathogens. It was found to have antifungal activity against *C. albicans* (25 μg/mL MIC, static effect) only. Similarly Heptaibin, an antifungal peptaibol antibiotic (against *C. albicans* with MIC 32 μg/mL) has been reported from *Emericellopsis lixii* BAUA8289 [67].

## Conclusions

Thus, present study describes a strain of *Trichoderma lixii* (IIIM-B4) which was isolated as an endophyte from *Bacopa monnieri* L. and showed the production of antifungal peptaibol. IIIM-B4, the only reported *Trichoderma,* as an endophyte of *Bacopa monnieri* has not yet been explored for neurological disorders, but recently a study suggested that a new cyclopentenone isolated from *Trichoderma* sp. with free radical scavenging properties, might be effective in Alzheimer’s disease (AD) models (Harrison 2012). Hence it can also be explored for neurological disorders.

## Additional file

Additional file 1: **Table S1.** Mycelia growth, Colony characters and sporulation pattern of B4 culture plate on different medium. **Table S2.** Antimicrobial activities of extract of *Trichoderma lixii* (IIIM-B4). Microorganisms used were *Bacillus subtilis, Pseudomonas aeruginosa*, *Salmonella typhimurium*, *Escherichia coli*, *Klebsiella pneumonia*, *Staphylococcus aureus*, *Candida albicans*. The lowest concentration at which there was no visible growth after 16 h was considered as minimum inhibitory concentration (MIC). **Table S3.** Comparative summation of novel Tribacopin AV and known sequences of 11 residue peptaibols produced by *Trichoderma lixii.* **Figure S1.** Mycelia growth, Colony characters and sporulation pattern of *Trichoderma lixii* (IIIM-B4) e*ndophytic fungi* on different medium (i) Potato Dextrose Agar (PDA) (ii) malt extract agar (iii) yeast extract malt extract agar (iv) Sabourauds dextrose agar (v) Oat meat agar (vi) Rose Bengal agar (vii) Potato carrot agar (viii) Corn meal agar (ix) Synthetic medium 1(x) synthetic medium 2. **Figure S2.** Mass studies depicting the peptaibols production from *Trichoderma lixii* (IIIM-B4) in different media. **Figure S2a)** Potato Dextrose Broth **Figure S2a’)** Potato Dextrose Agar **Figure S2b)** Malt extract agar **Figure S2c)** Yeast extract malt agar MEA **Figure S2d)** Sabourauds dextrose agar **Figure S2e)** Oat meat agar **Figure S2f)** Rose Bengal agar **Figure S2g)** Potato carrot agar **Figure S2 h)** Corn meal agar **Figure S2i)** Synthetic medium 1 **Figure S2j)** Synthetic medium 2. **Figure S3.** Separation of Group A peptaibols from Group C *from extract of Trichoderma lixii* through HPLC. **Figure S4.** Mass spectra of peptaibol Tribacopin AV having mass 1185. **Figure S5.** Sequence of Tribacopin AV based on MS/MS studies. (DOC 7700 kb)

## Abbreviations

ITS: Internal transcribed spacer
MIC: Minimum inhibitory concentration
