# Supplementary material for: Trichoderma lixii (IIIM-B4), an endophyte of Bacopa monnieri L. producing peptaibols
Source: BMC Microbiol. 2019 May 16;19:98. doi: 10.1186/s12866-019-1477-8 (PMC6524271; doi:10.1186/s12866-019-1477-8)

***Trichoderma lixii* (IIIM-B4), an endophyte of *Bacopa monnieri* L. producing peptaibols**

Meenu Katoch^1!*^, Deepika Singh^2!*^, Kamal K Kapoor^3^, RA Vishwakarma^4^

^1^Microbial Biotechnology Division, Indian Institute of Integrative Medicine, Jammu, India

^2^Quality Control and Quality Assurance Division, Indian Institute of Integrative Medicine, Jammu, India

^3^Department of Chemistry, University of Jammu, 180001, India

^4^Medicinal Chemistry Division, Indian Institute of Integrative Medicine, Jammu, India

**Table S1:** Mycelia growth, Colony characters and sporulation pattern of IIIM-B4 on different medium

| **Media** | **Colony diameter**  **(mm)** | **Texture** | **Surface colour** | **Reverse colour** | **Zonation** | **Sporulation** |
| --- | --- | --- | --- | --- | --- | --- |
| PDA | 90 | velvet | White velvet with greenish spores | Cream | none | Moderate |
| MEA | 90 | velvet | Thick yellowish velvet at centre and white at periphery | Brown colour | none | No |
| YMA | 90 | velvet | White velvet | Cream | none | Slight spores at periphery |
| SABD | 90 | velvet | White velvet | Cream | none | No |
| Oat meal agar | 86.0±3.3 | velvet | White velvet with greenish spores | Yellow colour | Concentric zone | Poor |
| Rose Bengal agar | 86.0±0.3 | Thick velvet | Creamish velvet with green spores | Pink colour | none | Heavily in centre and moderate at periphery |
| Potato carrot agar | 90.0±1.5 | Fine | Transparent with green spores | Colour less | With concentric zones | Moderate at periphery |
| Corn meal agar | 68.0±2.3 | Fine | Transparent with green spores | Colour less | none | Moderate all over the surface |
| Synthetic media 1 | 68.0±0.9 | Fine | Transparent with green spores | Colour less | none | Heavily in patches |
| Synthetic media 2 | 84.0±0.3 | Velvet thick | Creamish with green spores | Slightly yellow orange | none | Heavily at periphery |

**Table S2:** Antimicrobial activities of extract of *Trichoderma lixii* (IIIM-B4). Microorganisms used were *Bacillus subtilis, Pseudomonas aeruginosa*, *Salmonella typhimurium*, *Escherichia coli*, *Klebsiella pneumonia*, *Staphylococcus aureus*, *Candida albicans*. The lowest concentration at which there was no visible growth after 16 h was considered as minimum inhibitory concentration (MIC)

| **Pathogen** | Peptaibol mix of *Trichoderma lixii* (IIIM-B4) (µg/mL) | **Streptomycin/**  **Amphotericin B**  **(µg/mL)** | |
| --- | --- | --- | --- |
|  | MIC | **MIC** | **MBC** |
| *S. aureus* (MTCC No. 737) | >100 | 0.011 | 0.3125 |
| *B. subtilis* (MTCC No. 121) | >100 | 0.3125 | 0.3125 |
| *S.* *typhimurium* (MTCC No. 98) | >100 | 0.3125 | 0.3125 |
| *K. pneumonia* (MTCC No. 109) | >100 | 0.3125 | 0.3125 |
| *P. aeruginosa* (MTCC No. 424) | >100 | 0.3125 | 0.3125 |
| *E. coli* (MTCC No. 118) | >100 | 0.3125 | 0.3125 |
| *C. albicans* (MTCC No. 183) | 25 | 6.25 | 6.25 |

**Table S3:** Comparative summation of novel Tribacopin AV and known sequences of 11 residue peptaibols produced by *Trichoderma lixii*

|  | MW | 1 | 2 | 3 | 4 | 5 | 6 | 7 | 8 | 9 | 10 | 11 | Ref |
| --- | --- | --- | --- | --- | --- | --- | --- | --- | --- | --- | --- | --- | --- |
| 1 | 1203 | AcU | Q | Vx | Lx | U | P | Vx | Lx | U | P | Lxol | Mikkola et al. (2012) |
| 2 | 1197 | AcU | Q | Lx | Lx | U | P | Lx | Lx | U | P | Vxol | Rohrich et al. (2014) |
| 3 | 1197 | AcVx | Q | Lx | Lx | U | P | Vx | Lx | U | P | Vxol | Rohrich et al. (2014) |
| 4 | 1197 | AcVx | Q | Lx | Vx | U | P | Lx | Lx | U | P | Vxol | Rohrich et al. (2014) |
| 5 | 1197 | AcVx | Q | Vx | Lx | U | P | Lx | Lx | U | P | Vxol | Rohrich et al. (2014) |
| 6 | 1197 | AcU | Q | Lx | Lx | U | P | Vx | Lx | U | P | Lxol | Rohrich et al. (2014) |
| 7 | 1197 | AcU | Q | Lx | Vx | U | P | Lx | Lx | U | P | Lxol | Rohrich et al. (2014) |
| 8 | 1197 | AcU | Q | Lx | Lx | U | P | Lx | Lx | U | P | Lxol | Rohrich et al. (2014) |
| 9 | 1197 | AcVx | Q | Vx | Lx | U | P | Vx | Lx | U | P | Lxol | Rohrich et al. (2014) |
| 10 | 1197 | AcVx | Q | Lx | Vx | U | P | Vx | Lx | U | P | Lxol | Rohrich et al. (2014) |
| 11 | 1197 | AcVx | Q | Vx | Vx | U | P | Lx | Lx | U | P | Lxol | Rohrich et al. (2014) |
| 12 | 1185 | AcG | L | L | L | A | L | P | L | U | V | Q-OH | Present study |
| 13 | 1155 | AcU | N | Vx | Vx | U | P | Vx | Lx | U | P | Lxol | Mikkola et al. (2012) |
| 14 | 1155 | AcU | N | Vx | Vx | U | P | Lx | Lx | U | P | Vxol | Mikkola et al. (2012) |
| 15 | 1155 | AcU | N | Vx | Vx | U | P | Lx | Vx | U | P | Lxol | Mikkola et al. (2012) |
| 16 | 1161 | AcU | Q | Vx | Vx | U | P | Lx | Lx | U | P | Lxol | Mukherjee et al. (2011) |
| 17 | 1169 | AcU | N | Lx | Vx | U | P | Lx | Lx | U | P | Vxol | Mikkola et al. (2012) |
| 18 | 1169 | AcU | N | Lx | Vx | U | P | Vx | Lx | U | P | Lxol | Mikkola et al. (2012) |
| 19 | 1169 | AcU | N | Vx | Lx | U | P | Lx | Lx | U | P | Vxol | Mikkola et al. (2012) |
| 20 | 1169 | AcU | N | Vx | Lx | U | P | Vx | Lx | U | P | Lxol | Mikkola et al. (2012) |
| 21 | 1175 | AcU | Q | Vx | Lx | U | P | Lx | Lx | U | P | Lxol | Mukherjee et al. (2011) |
| 22 | 1175 | AcU | Q | Lx | Lx | U | P | Vx | Lx | U | P | Lxol | Mukherjee et al. (2011) |
| 23 | 1183 | AcU | N | Lx | Lx | U | P | Lx | Lx | U | P | Lxol | Mikkola et al. (2012) |
| 24 | 1183 | AcU | N | Lx | Lx | U | P | Lx | Vx | U | P | Lxol | Mikkola et al. (2012) |
| 25 | 1183 | AcU | N | Lx | Lx | U | P | Vx | Lx | U | P | Lxol | Mikkola et al. (2012) |
| 26 | 1183 | AcU | N | Lx | Vx | U | P | Lx | Lx | U | P | Lxol | Mikkola et al. (2012) |
| 27 | 1183 | AcU | N | Lx | Lx | U | P | Lx | Lx | U | P | Lxol | Mikkola et al. (2012) |
| 28 | 1189 | AcU | Q | Lx | Lx | U | P | Lx | Lx | U | P | Lxol | Mukherjee et al. (2011) |
| 29 | 1211 | AcU | Q | Lx | Lx | U | P | Lx | Lx | U | P | Lxol | Mikkola et al.(2012) |

**Fig S1** Mycelia growth, Colony characters and sporulation pattern of *Trichoderma lixii* (IIIM-B4) endophytic fungi on different medium (i) Potato Dextrose Agar (PDA) (ii) malt extract agar (iii) yeast extract malt extract agar (iv) Sabourauds dextrose agar (v) Oat meat agar (vi) Rose Bengal agar (vii) Potato carrot agar (viii) Corn meal agar (ix) Synthetic medium 1(x) synthetic medium 2.


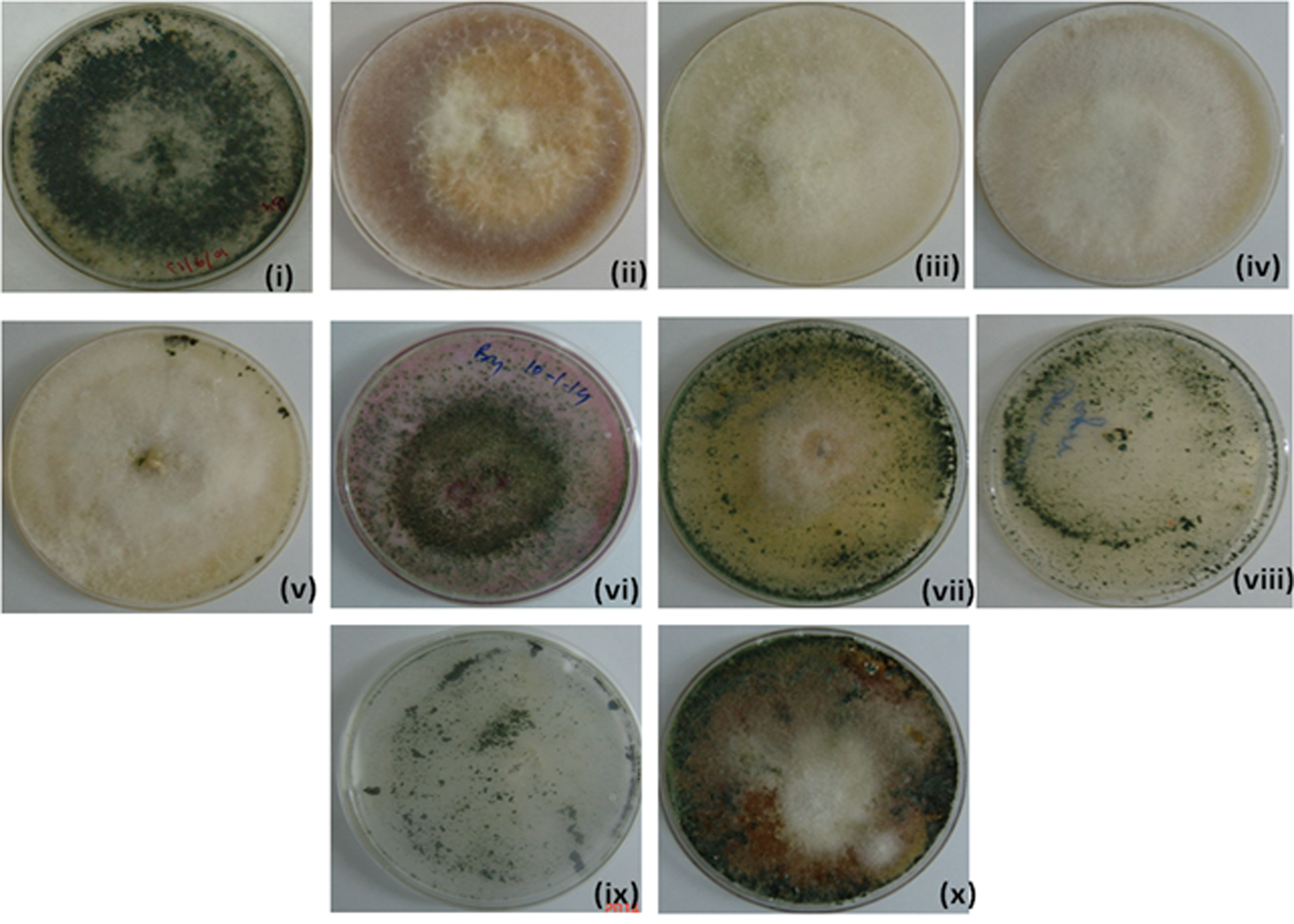


**Figure S2** Mass studies depicting the peptaibols production from *Trichoderma lixii* (IIIM-B4) in different media. **S2a)** Potato Dextrose Broth **S2a’)** Potato Dextrose Agar **S2b)** Malt extract agar **S2c)** Yeast extract malt agar MEA **S2d)** Sabourauds dextrose agar **S2e)** Oat meat agar **S2f)** Rose Bengal agar **S2g)** Potato carrot agar **S2h)** Corn meal agar **S2i)** Synthetic medium 1 **S2j)** Synthetic medium 2


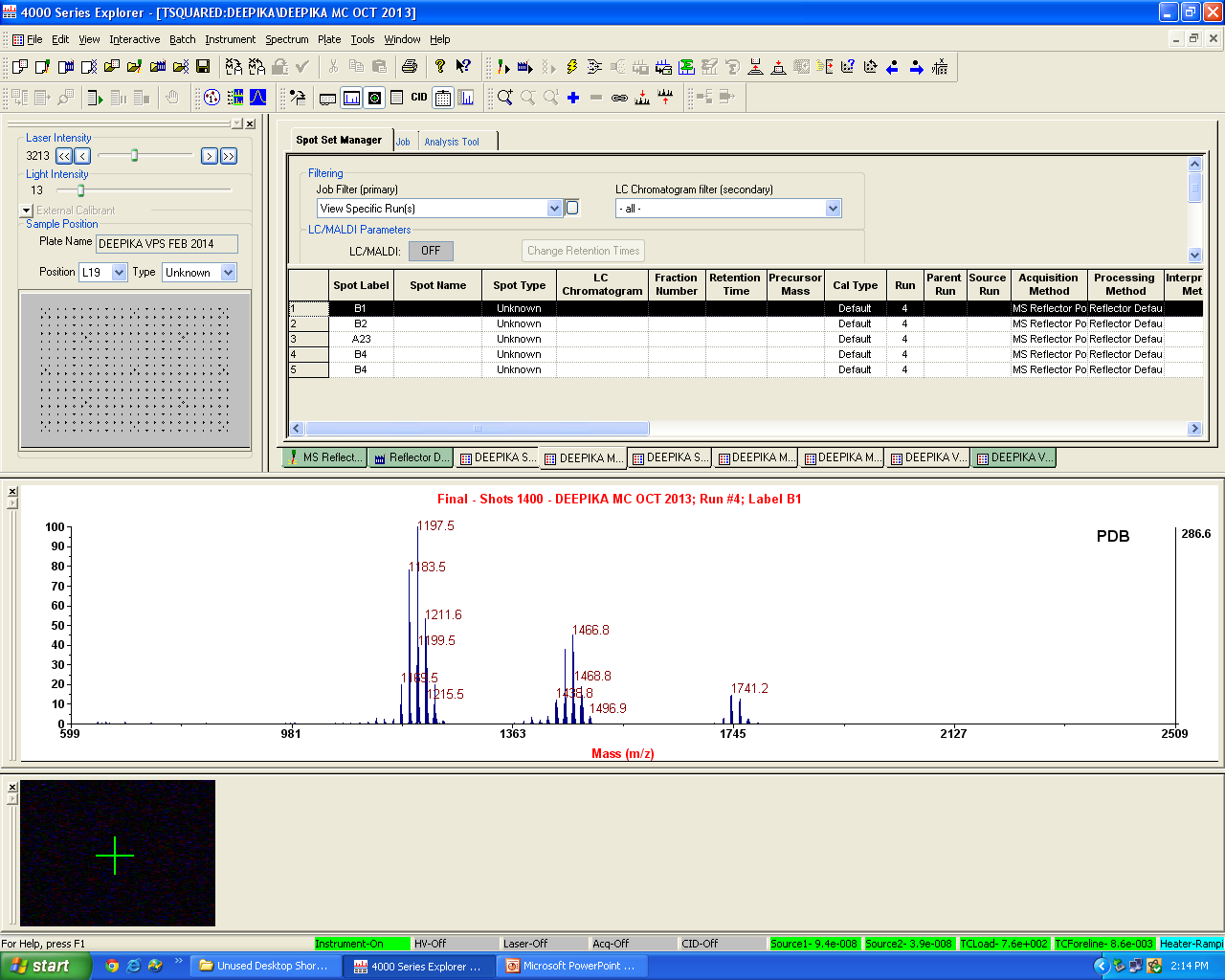


Figure S2(a)


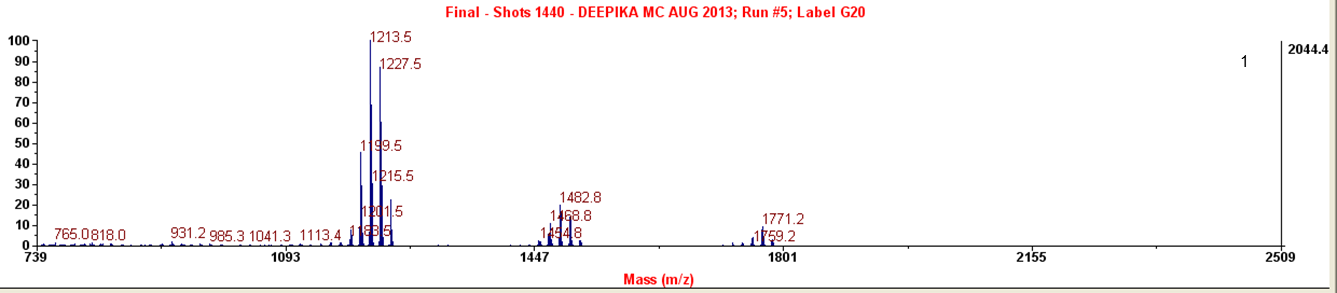


Figure S2(a’)


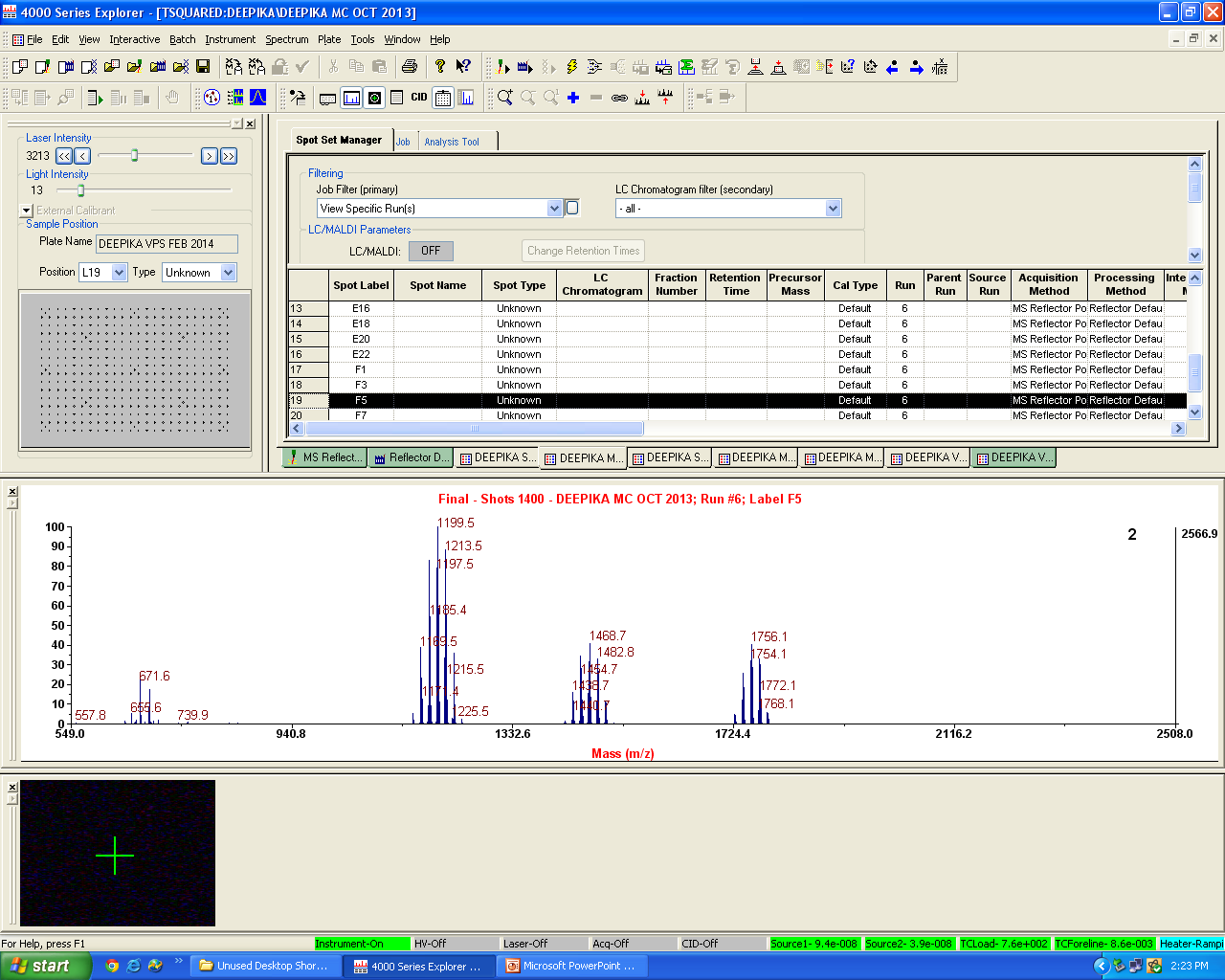


Figure S2(b)


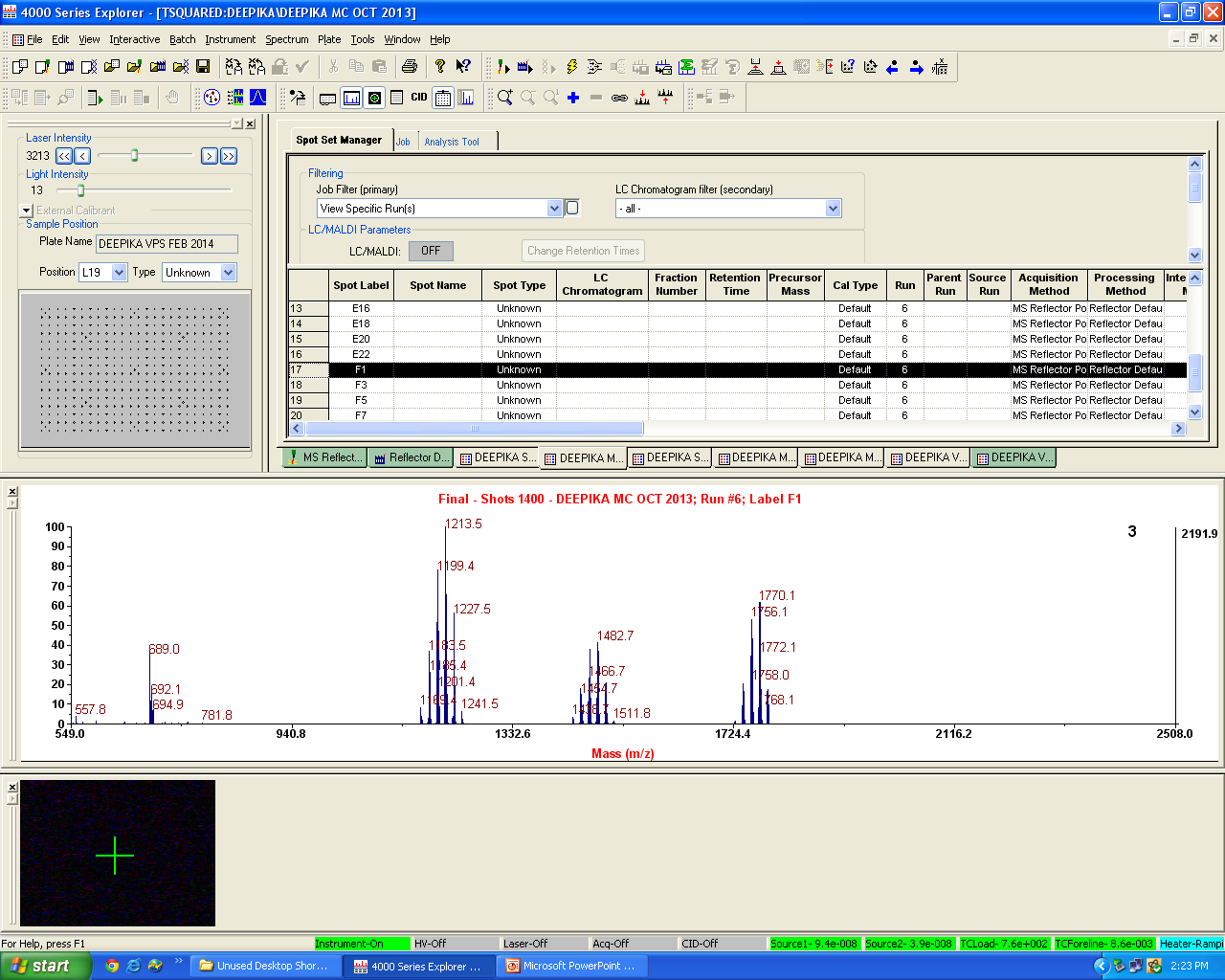


Figure S2(c)

**
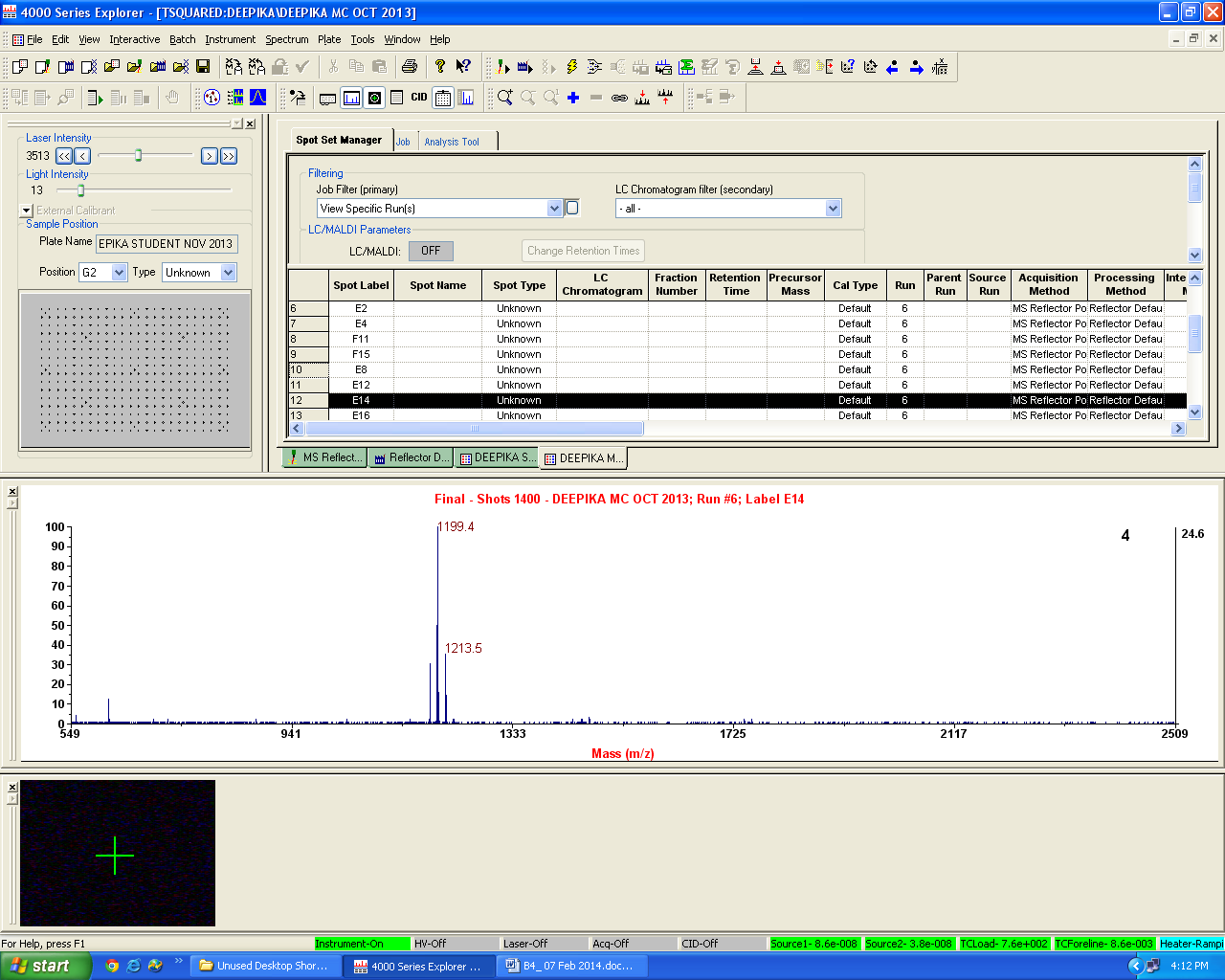
**

Figure S2(d)


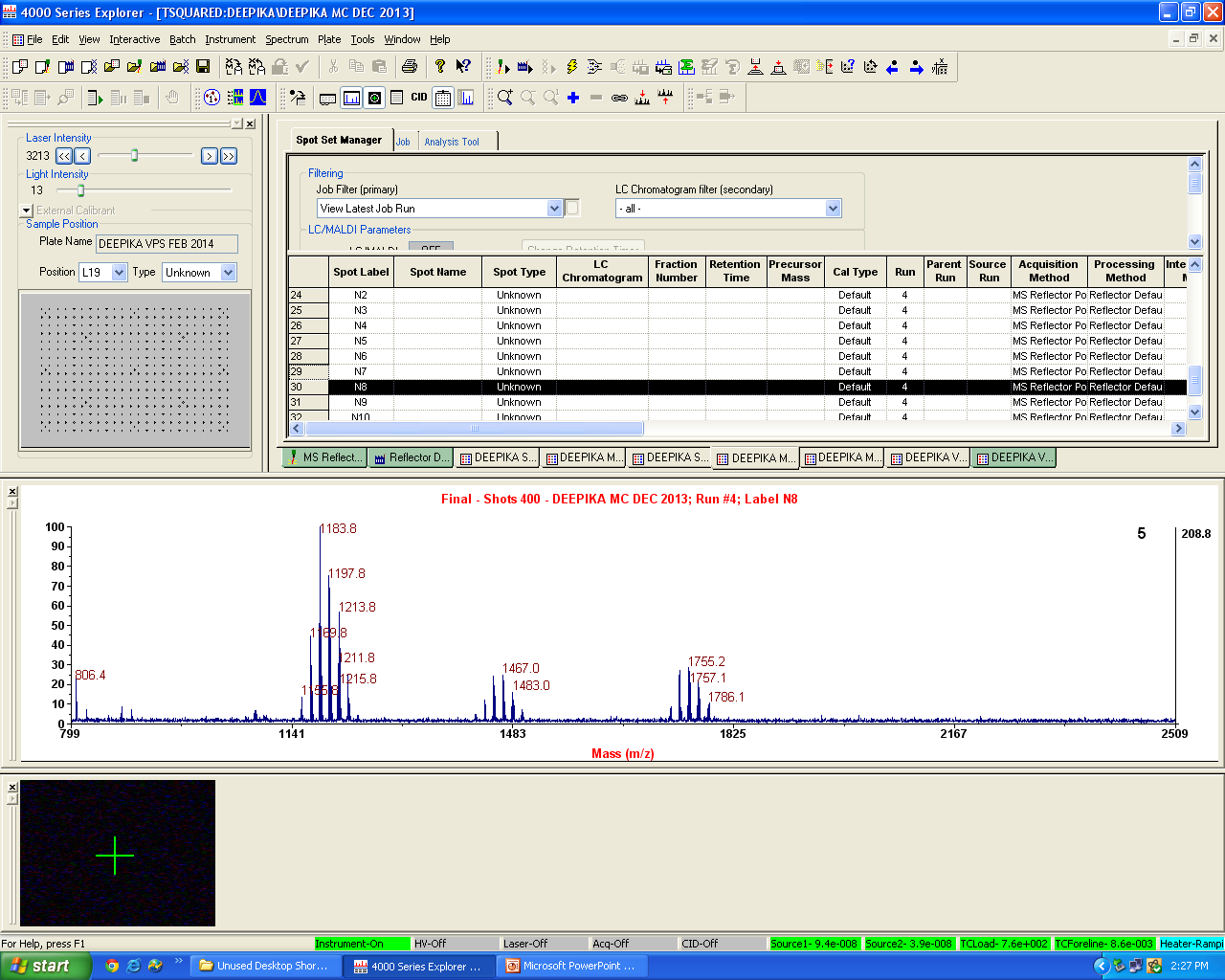


Figure S2(e)


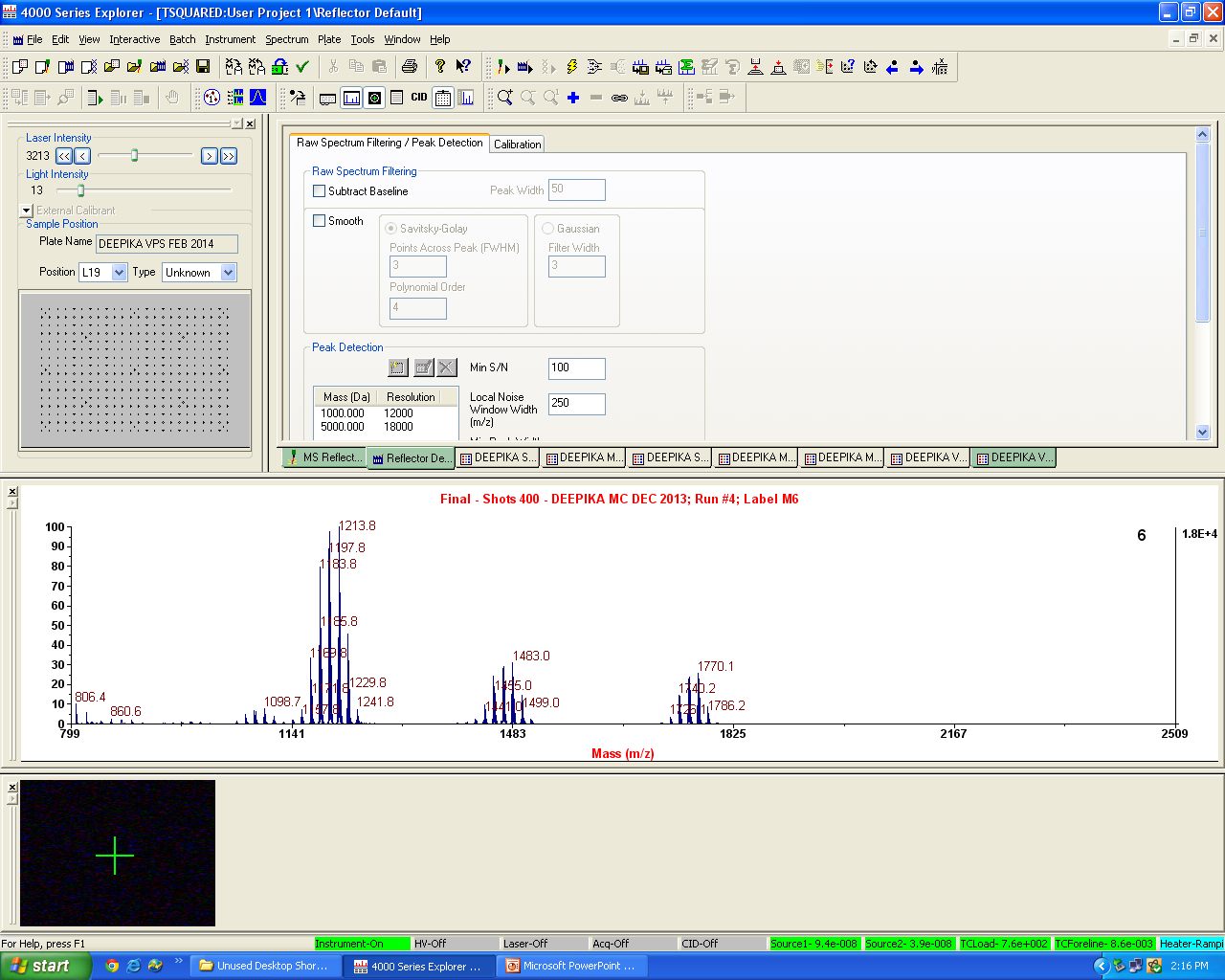
Figure S2(f)


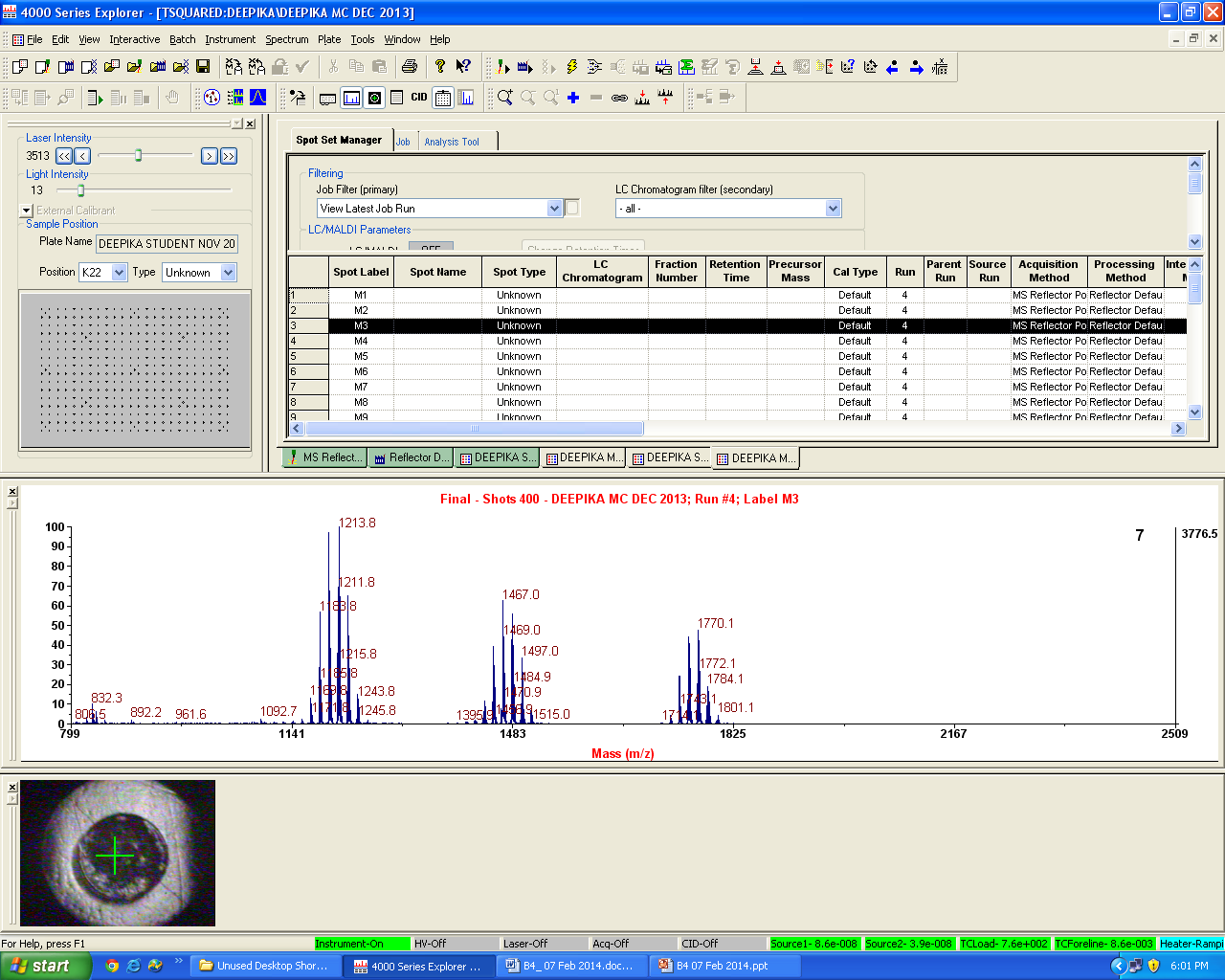


Figure S2(g)


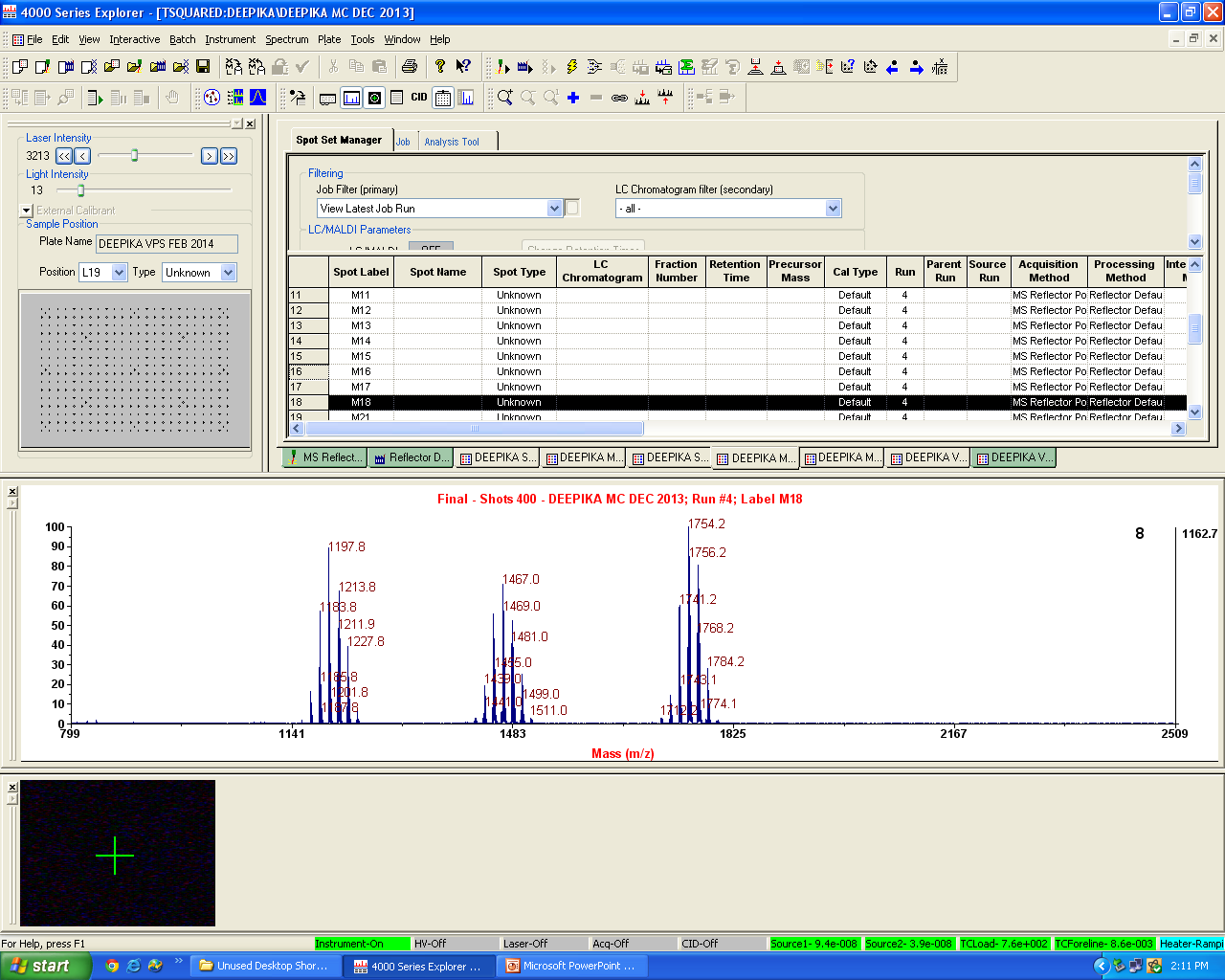


Figure S2(h)


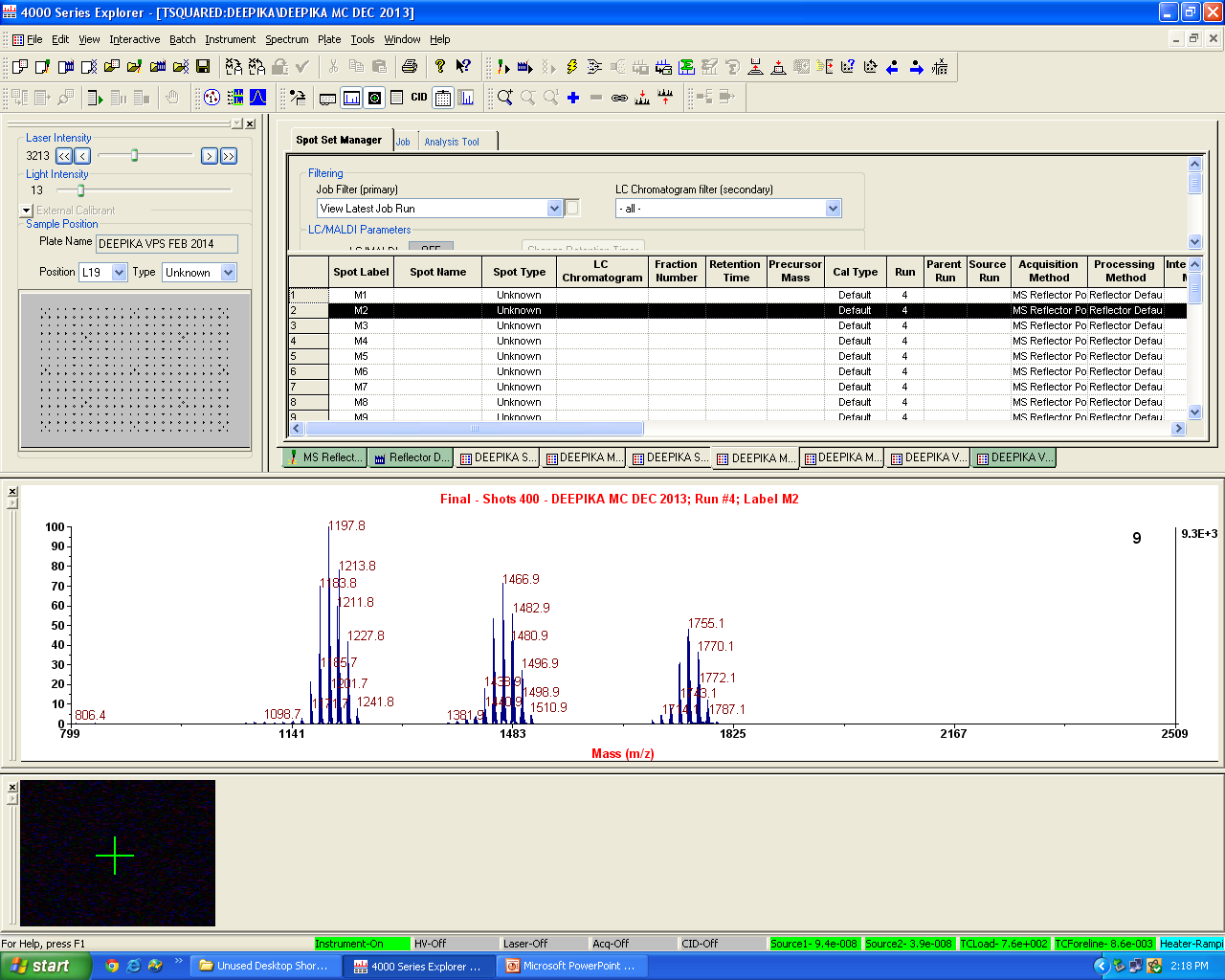


Figure S2(i)


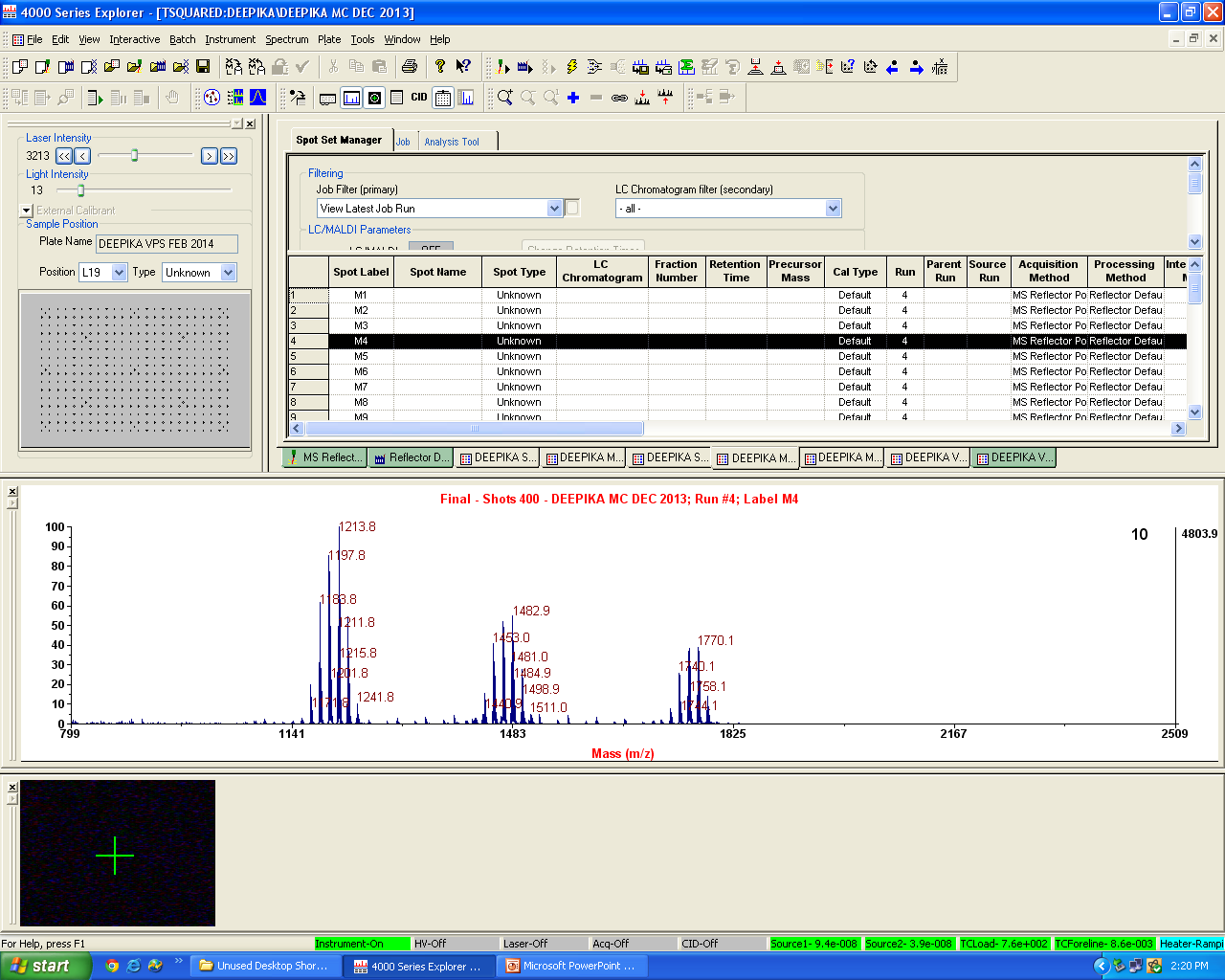


Figure S2(j)

**Fig S3** Separation of Group I peptaibols from Group III from extract of *Trichoderma lixii* through HPLC


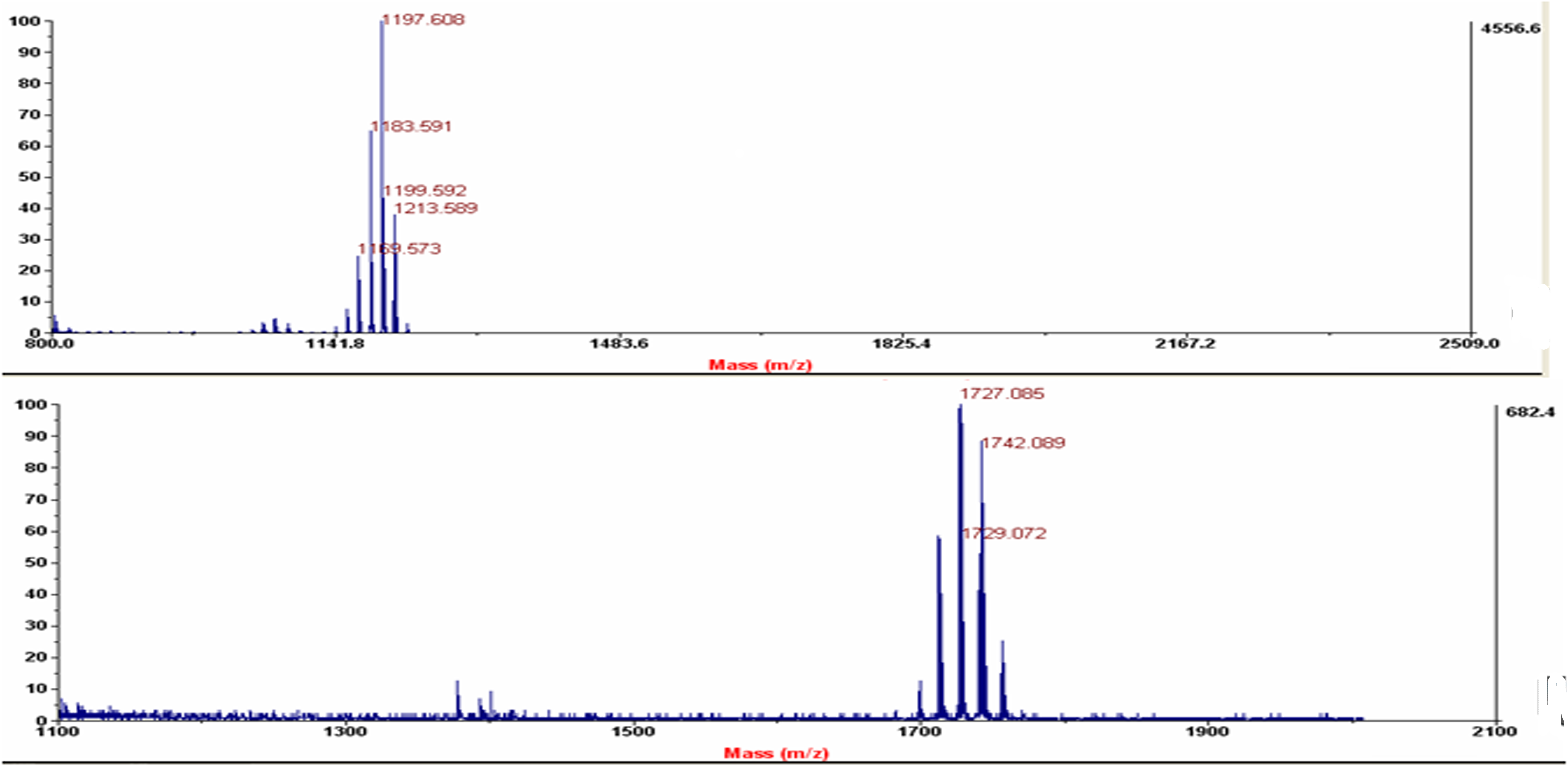


**Fig S4** Mass spectra of purified peptaibols Tribacopin AV having mass 1185

**Fig S5** Sequence of Tribacopin AV based on MS/MS studies


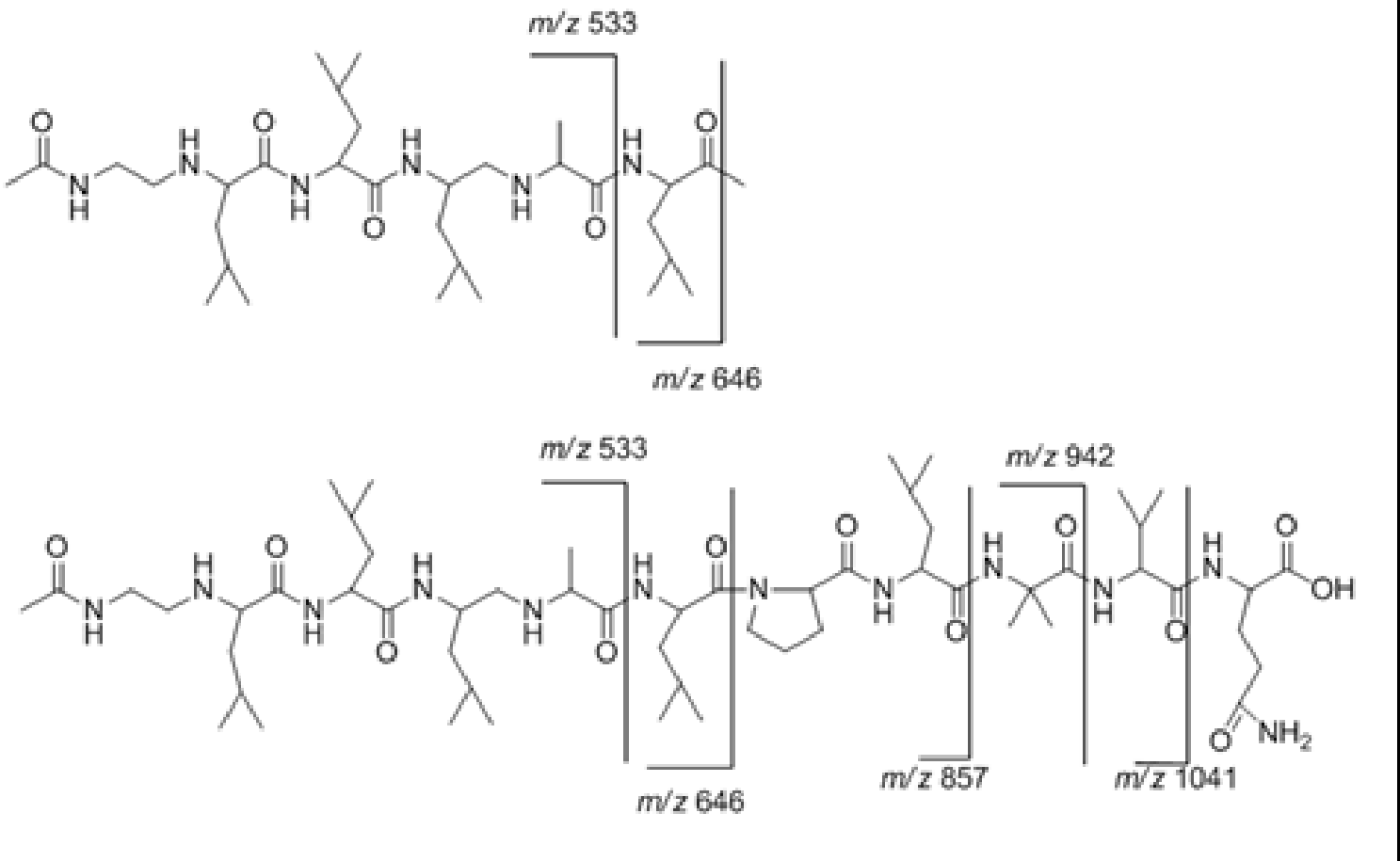

Supplement: Supplementary file 1 — Table S1. Mycelia growth, Colony characters and sporulation pattern of B4 culture plate on different medium. Table S2. Antimicrobial activities of extract of Trichoderma lixii (IIIM-B4). Microorganisms used were Bacillus subtilis, Pseudomonas aeruginosa, Salmonella typhimurium, Escherichia coli, Klebsiella pneumonia, Staphylococcus aureus, Candida albicans. The lowest concentration at which there was no visible growth after 16 h was considered as minimum inhibitory concentration (MIC). Table S3. Comparative summation of novel Tribacopin AV and known sequences of 11 residue peptaibols produced by Trichoderma lixii. Figure S1. Mycelia growth, Colony characters and sporulation pattern of Trichoderma lixii (IIIM-B4) endophytic fungi on different medium (i) Potato Dextrose Agar (PDA) (ii) malt extract agar (iii) yeast extract malt extract agar (iv) Sabourauds dextrose agar (v) Oat meat agar (vi) Rose Bengal agar (vii) Potato carrot agar (viii) Corn meal agar (ix) Synthetic medium 1(x) synthetic medium 2. Figure S2. Mass studies depicting the peptaibols production from Trichoderma lixii (IIIM-B4) in different media. Figure S2a) Potato Dextrose Broth Figure S2a’) Potato Dextrose Agar Figure S2b) Malt extract agar Figure S2c) Yeast extract malt agar MEA Figure S2d) Sabourauds dextrose agar Figure S2e) Oat meat agar Figure S2f) Rose Bengal agar Figure S2g) Potato carrot agar Figure S2 h) Corn meal agar Figure S2i) Synthetic medium 1 Figure S2j) Synthetic medium 2. Figure S3. Separation of Group A peptaibols from Group C from extract of Trichoderma lixii through HPLC. Figure S4. Mass spectra of peptaibol Tribacopin AV having mass 1185. Figure S5. Sequence of Tribacopin AV based on MS/MS studies. (DOC 7700 kb) [file 12866_2019_1477_MOESM1_ESM.doc]
